# Solid-state hydrogen storage in metal hydrides: mechanistic insights into kinetics, activation, and destabilization strategies

**DOI:** 10.1039/d6ra05276b

**Published:** 2026-07-21

**Authors:** Muhammad Nouman Qayyum, Abdul Majid, Muhammad Tayyab Raza

**Affiliations:** a Department of Physics, Hafiz Hayat Campus, University of Gujrat Gujrat Pakistan abdulmajid40@yahoo.com; b Department of Physics, Gachon University 1342 Seongnam-daero, Sujeong-gu Seongnam-si Gyeonggi-do South Korea tayyabraza464@gmail.com

## Abstract

In a hydrogen-based economy, safe and efficient hydrogen storage is a technological challenge for widespread hydrogen use. The liquid and compressed gas hydrogen storage techniques have some limitations that don't meet future demands. Solid-state materials are promising alternatives that can store hydrogen through physical or chemical binding at the material's surface, and they offer many advantages over conventional hydrogen storage systems. For the enhancement of hydrogenation characteristics of metal hydride systems, much research has been done. The recent advancements and various aspects of metal hydrides, including their characteristics and performance relevant to storage applications, are explored in this review article. Specifically, it examines a material's response to variation in temperature and pressure, and also provides understanding of reaction kinetics, cyclic stability, potential toxicity and hydrogen storage capacity. Specific emphasis is placed on the thermodynamic destabilization strategies that have been effective in achieving a rise in desorption plateau pressures, to enable hydrogen release at ambient temperature. In addition, the detailed mechanisms for how surface fluorination and catalysts are able to lower high activation energy barriers to rapidly facilitate absorption/desorption kinetics are carefully examined. The review also focuses on key engineering challenges such as degradation of structure and lattice strain arising from long-term cycling stability. The principle of H_2_ storage in metal hydride-based solid state systems and their applications in H_2_ technologies and advancing energy are also discussed herein.

## Abstract 2

In a hydrogen-based economy, safe and efficient hydrogen storage is a technological challenge for widespread hydrogen use.

## Graphical abstract

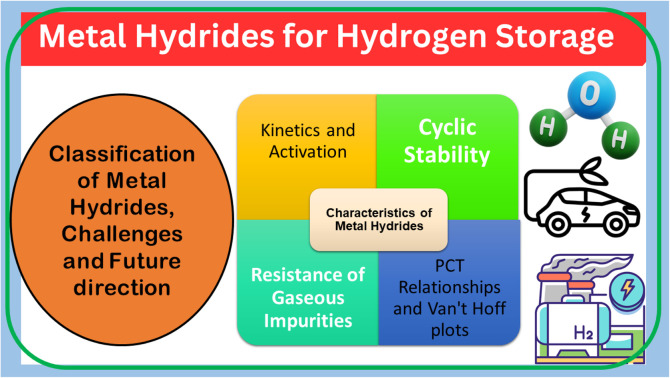

## 1. Introduction

Hydrogen is regarded as a promising energy carrier for mobile and stationary applications owing to its ability to minimize environmental impacts and reduce dependence on imported fossil fuels, especially in resource-limited countries.^1,2^ One of the major obstacles in the development of the hydrogen economy is the efficient storage of hydrogen. It can be stored as compressed gas, liquefied hydrogen at cryogenic temperatures, or in solid form *via* chemical or physical bonding with materials including metal hydrides, complex hydrides, and carbon materials. Each of these storage methods exhibits distinct advantages that make them attractive for the storage of H_2_.^3,4^ Current storage technologies allow the storage of hydrogen in either compressed or liquefied form using pressurised or cryogenic storage systems.^2^ Traditional hydrogen storage methods are challenging because gaseous phase hydrogen has a very low density and boiling point at atmospheric pressure. Additionally, maintaining hydrogen in the form of liquid form requires continuous refrigeration to sustain cryogenic temperatures.^5,6^

High pressure is needed for H_2_ storage in gaseous form, which can be dangerous.^7^ Metal hydrides have the ability to store hydrogen in a solid form at lower pressures, making them much more stable. Metal hydrides can store large amounts of hydrogen in a compact lattice because they possess a superior volumetric energy density.^8^ Furthermore, these solid-state materials do not require high-pressure compression or cryogenic liquefaction, which minimizes the risks which are associated with containment failures. That is why they are better than other mechanical storage techniques. Metal hydrides store hydrogen at ambient conditions, making them superior for fuel cell applications.^8–10^ The release of hydrogen from a material's surface is an endothermic process because heat is required to restore hydrogen; any system failure or loss of thermal energy can cause self-limiting discharge, which increases the risk of uncontrolled, rapid leakage. The desorption of the hydrogen in these materials takes place by changing thermodynamic equilibrium, which is achieved through isothermal pressure reduction or isobaric thermal activation.^11–13^

When hydrogen reacts with metals, metal hydrides form, which possess high hydrogen storage capacities. They are suitable for fuel cell vehicles because they offer higher hydrogen storage capacity than liquid or compressed gas systems.^14^ These materials are divided into three categories: covalent, ionic and metallic. In this review article, the general characteristics of these materials, such as kinetics, activation, and PCT relationships, are also discussed. The rates of adsorption and desorption are affected by temperature and particle size, as described by kinetics.^15^ For the removal of surface impurities and the enhancement of hydrogen adsorption and activation processes, mechanical and thermal treatments are important. Whereas, information about entropy, enthalpy, and hydrogen storage capacity is provided by PCT curves.^16,17^ Although metal hydrides have the potential to store hydrogen, there are some challenges in their practical implementation, including poor cyclic stability, high desorption enthalpies, and sluggish sorption kinetics.^16,17^ Current materials science research is focusing on modelling metal hydrides with destabilised thermodynamics and improved reaction rates to achieve high storage capacities. The goal set by the U.S. Department of Energy for the development of hydrogen storage media for mobile hydrogen applications has not yet been achieved.^18^

Recent research has focused on modifying their cyclic stability, enhancing kinetics and decreasing desorption temperatures.^19^ Many suitable catalysts, and the ball-milling process, modify surface characteristics and introduce structural defects, which improve kinetics. Metal hydrides are better for storing hydrogen, but still have problems.^20,21^ There is a need to improve their cyclic hydrogenability and to increase the storage capacity of these materials. Mg-based metal hydrides are considered to be better because their storage capacity is about 7.7 wt%.^22^ We can increase hydrogen storage cycles, remove impurities, and increase hydrogen storage capacity by improving the general properties of metal hydrides. One more challenge in these systems is that they are heavy, and for transportation and daily use, metal hydrides should have a low weight; this problem must be addressed in the future.

## 2. Metal hydrides

Chemical compounds which are formed when hydrogen reacts with the metals are called metal hydrides.^23^ These compounds are widely used in materials science and energy storage applications. In metal hydrides, hydrogen is chemically bonded to metal alloys or metals. In the hydrogen absorption process, hydrogen initially dissociates upon reaching the metal surface and diffuses into the metal lattice.^24,25^ The storage of the hydrogen in metal hydrides is suitable because they can absorb large amounts of H_2_ within a crystal lattice. They can store hydrogen more than the liquid and compressed gas H_2_ storage systems. It is very easy to adsorb and desorb hydrogen in metal hydrides for this reason they are consider better for hydrogen-powered automobiles and fuel cells.^26^ The storage of hydrogen in these solid-state systems are safer than other bulky cylindrical systems which are used for liquid and compressed hydrogen gas. Most importantly, they are light-weight compared to other bulky systems and are very suitable for portable applications.^27,28^ Many factors are essential for the selection of metal hydride for the storage of hydrogen (H_2_). The synthesis of hydrides and breakdown processes should be reversible at operating temperatures and pressures relevant to the application.^29^ Furthermore, the material must possess high reversible H_2_ storage capacity under operational conditions. These features are dictated by the pressure-composition-temperature (PCT) parameters in H_2_ gas systems that include hydride-forming materials, where the reversible storage ability corresponds with the plateau width on the isotherm of pressure-composition.^30^

**Fig. 1:**
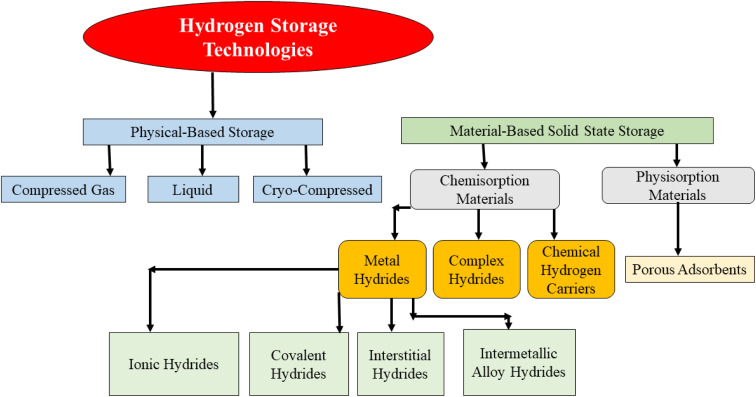
Schematic classification of physical- and materials-based hydrogen storage technologies.

Fig. 1 shows an overview of hydrogen storage technologies, which can be classified into physical-based storage technologies and materials-based (solid-state) storage technologies.^31,32^ Physical-based storage is based on the compression or cooling of molecular hydrogen by compressed gas, liquid hydrogen or cryo-compressed hydrogen without any chemical reaction.^33^ In the materials-based approach, a host material is used to store hydrogen and can be divided into physisorption materials, which store hydrogen chemically *via* weak van der Waals forces at low temperature, and chemisorption materials which bind hydrogen through a chemical bond, typically by heating. Chemisorption materials are then classified according to the type as metal hydrides, complex hydrides and chemical hydrogen carriers, which have different storage density, operating temperature and reversibility characteristics.^34^ We can classify all metal hydrides according to the character of the metal–hydrogen bond, *e.g.*, ionic (saline) hydrides, covalent hydrides, interstitial (metallic) hydrides and intermetallic/alloy hydrides, which determine their storage capacity, their thermal stability and their desorption behavior.

The absorption of H_2_ (hydrogenation) and H_2_ desorption process (dehydrogenation) are dependent upon the relation between the current pressure of H_2_ and the plateau pressure at the prevailing temperature.^35,36^ The hysteresis and plateau slope of the PCT characteristics of many efficient systems are crucial for H_2_ compression applications, as they substantially reduce the ratio of compression and efficiency of process within the specified temperature range.^36,37^ Weight percentage (wt%) of the system is calculated to estimate that how much H_2_ is stored in the material. The characteristics of the PCT graphs can be determine with the help of process of hydrogenation. When the amount of H_2_ increases, more diffusion of H_2_ through the metal hydride lattice occurs, and more interstitial sites are occupied.^8^

## 3. Classification of metal hydrides

Metal hydrides possess a wide range of different properties and structures. It is essential to classify them on the basis of their structural characteristics and the nature of the bond. This section discusses the key features of different metal hydrides. Hydrogen can form binary compounds with many other elements except He, Ne, Ar, Kr, Pm, Os, Ir, Rn, Fr, and Ra.^38–41^ We can classify the metal hydrides into different categories based on the bond between specific elements and hydrogen. The complete distinction between metal hydrides is not possible because we do not entirely understand the nature of the bond within them. However, they are classified as ionic hydrides or saline hydrides, covalent hydrides or molecular hydrides, interstitial hydrides or metallic hydrides, and metal hydride alloys.^41,42^

### 3.1. Ionic hydrides and covalent hydrides

Ionic hydrides are formed when the alkali and alkaline earth metals, which are electropositive, react with the hydrogen.^43^ Ionic hydrides are solid crystalline substances, and in these compounds, hydride ion (H^−^) is present, and positively charged metal forms an ionic bond with H^−^. Some examples of ionic hydrides are lithium, calcium, and sodium hydrides.^39,44^ In these materials, hydrogen gains an electron from metal, and the ionic bond forms between them. Covalent hydrides are formed when H_2_ reacts with the elements of group 12 to group 17, and the formation of these compounds occurs when a covalent bond is formed between H_2_ atoms and elements of these groups, which means electron pairs are shared in this process.^45,46^ Polymeric hydrides often form when group 12 and 13 elements react with hydrogen. The hydrides formed with elements from group 14 to group 17 are volatile.^47^

### 3.2. Metallic hydrides

Metallic hydrides are formed when the hydrogen atom occupies the interstices within the crystal lattice of the transition metals, lanthanides, and actinides.^48^ These are highly metallic and non-stoichiometric, meaning their composition can vary. In these hydrides, hydrogen is bonded in octahedral or tetrahedral spaces within the lattice. That is the reason when distortion occurs, their metallic nature does not change.^35,39,49^ The hydrides that possess strong ionic or covalent character are also named non-interstitial hydrides, and they show high H_2_ storage wt%, and they are also more stable than interstitial hydrides. So, the restoration of the H_2_ happens at high temperatures in these materials.^42^ The atomic weight of the elements of the ionic hydrides and covalent hydrides is lower than the metallic hydride. Also, the ionic and covalent bonds are stronger in these hydrides than metallic bonds, which are present in metallic hydrides. So, they are beneficial for storing more H_2_ molecules and releasing H_2_ molecules at high temperatures without any distortion in structure.^50–53^

### 3.3. Metal hydride alloys

Complex material or an alloy can be used only for the formation of the metal hydrides.^54^ Alloys that form metal hydrides are of three types: high entropy alloys, solid solutions, and intermetallic compounds.^55^ Two elements, A and B, are used for the formation of the intermetallic compounds, and they are in stoichiometric ratio, A elements can form stable hydrides, while B elements form unstable hydrides. When the distribution of one or more solutes in the interstitial site of the crystal lattice takes place, then solid solutions are made and without any stoichiometric ratio this process takes place.^48,56,57^

Unlike conventional alloys, which are made from one or two elements, high entropy alloys (HEAs) are made up of five or more than five different elements that are present in same or high proportions.^58,59^ The distinctive composition of the elements increases the entropy of mixing. They have high thermal stability and more interstitial sites available for the hydrogen due to their complex arrangement, which increases the hydrogen storage capacity of the HEAs.^55,60^ Lundin *et al.* demonstrated that the properties of metal hydrides and formation enthalpy depend on the interstitial sites. The hydride is more stable if the interstitial sites are bigger.^61,62^

## 4. General characteristics of metal hydrides

Metal hydrides possess many characteristics that control their behaviour and on which their applications depend. The reactivity and stability of these materials depend on the variations in the bonding types. The structure of metal and the composition of the elements play a very important role in reversible hydrogen absorption and desorption. Hydrogen storage capacity and kinetics are mostly related to the metal's crystal lattice and composition. For the determination of operating temperature, the thermal stability of the material is very necessary. The reactivity of metal hydride with moisture and air impacts the practical usage.

For hydrogen storage, the essential characteristics of metal hydrides will be explained in detail in this section. The characteristics that determine their appropriateness kinetics, activation, PCT relationships, impurity resistance, cycle stability, and safety will be explored.

### 4.1. Kinetics

The system's kinetics defines the speed at which absorption of hydrogen occurs in the metal hydrides and released from them.^54^ This factor is very important for using metal hydrides for portable power sources or fuel cell applications.^63^ For the recharging and refueling fast absorption kinetics are essential for the material. Many factors affect the absorption kinetics: intrinsic diffusion rate of hydrogen in metal's crystal lattice, presence of impurities, surface of metal's crystal lattice, presence of impurities, surface of metal and pressure and temperature of hydrogen gas.^23,51,54^

It is very important to understand the desorption kinetics if we want to know how fast the hydrogen is released from metal hydride.^64^ During the generation of power in fuel cells, it is necessary to deliver hydrogen on demand, and this can be possible if we understand the desorption kinetics of the materials.^65,66^ Many factors affect the desorption kinetics of the materials, including metal–hydrogen bond strength, heat transfer properties of metals, temperature of metals, and pressure surrounding the environment.^64,67^ Metal hydrides with good kinetics can quickly store and restore the hydrogen.^51^ The material would be less useful when it possessed slow kinetics, which results in prolonged refuelling or delayed hydrogen supply.^57^

The investigation of absorbed H_2_ molecules hydriding/dehydriding (H/D) kinetics is essential for practical applications. Puszkiel proposed a general technique for evaluating H/D kinetics.^68^ The rate of absorption and desorption depends upon the morphology, pressure, and temperature, the eqn (1) describes the relation between them:1
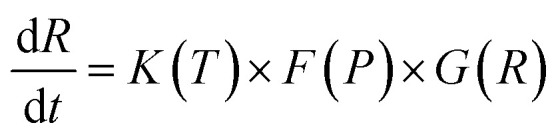
Here, *R* represents the proportion of hydrogen reacted to the material, *K*(*T*) denotes the term which depends on the temperature, *F*(*P*) signifies the term which depends on the pressure, and *G*(*R*) represents the term which depends on morphology. The dependence of the *K*, *F*, and *G* functions on temperature, pressure, and morphology (regarding the percentage of hydrogen) may be examined separately. To this end, eqn (2) is helpful:2
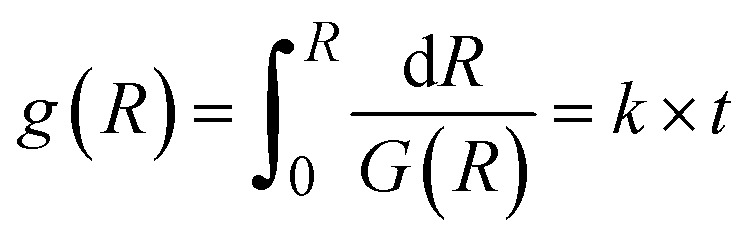


Eqn (2) shows the integral of 
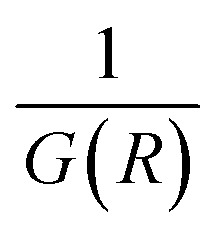
 w.r.t *R* having limit 0 to *R*. This eqn (2) links the H_2_ proportion (*R*) with time (*t*), *k* depends on pressure and temperature, and morphological effects are represented by *G*(*R*). Here, *k* represents the kinetic constant, and *k* can also be written as per eqn (3):

3 *k* = *K*(*T*) × *F*(*P*)

For *g*(*R*), a solid-state model must fully describe the *G*(*R*) function.^69^ The literature has several models, each explaining a specific process stage. Combining numerical and experimental data to identify the optimal model for describing the material's H/D process under specific circumstances is possible. The reaction rate dependency on temperature belongs to an Arrhenius eqn (4), assuming constant pressure:4
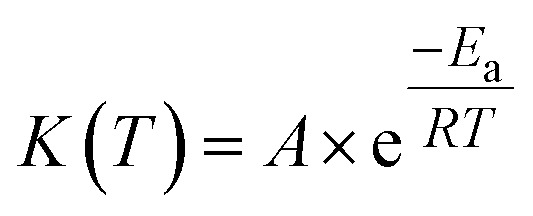


This equation tells that temperature depends on the rate constant for a chemical reaction. According to this, with the increase of temperature, the rate constant increases exponentially, relating it to the activation energy required for a reaction. Here, *A* represents a factor that depends on frequency, which tells us about the collision's rate among reactants, whereas *E*_a_ signifies the activation energy. The values of *A* and *E*_a_ may be determined from the fit of isothermal assessment obtained when the pressure is constant. It is essential to highlight the significance of the experimental study, based on the existing literature, which suggests that experimental conditions may impact kinetics.

Decreasing the size of the particles of metal hydrides to nanoscale surface area increases, enhancing the material's kinetics.^70^ Diffusion pathways can be shortened within metal hydrides at the nano level. By forming the alloy of metals with different elements, modifications in structural and electronic properties can be made, improving the kinetics.^8,70^ The addition of a catalyst is also another way to enhance the kinetics in the case of metal hydrides.^71^ The modification can increase the kinetics of complex hydrides.^54^ Complex hydrides are salts with hydrogen atoms in the anions.^52^ The sodium alanate (NaAlH_4_) kinetics have been improved for hydrogen storage by doping transition metal titanium (Ti) into the structure of NaAlH_4_.^72^ The strong bonds within NaAlH_4_ have been broken from this doping process, and the system's kinetics have been enhanced.

### 4.2. Activation

Activation is the process in which material is prepared for better reversible hydrogen absorption and desorption.^69^ This is necessary because when metal hydrides are prepared and exposed to air, the surface of those materials has oxides or many other contaminants that reduce hydrogen absorption.^73^ By heating the metal hydrides under a controlled atmosphere or vacuum, we can improve the microstructure of the materials and remove impurities from the surface.^74^ Thermal activation can also form many active sites, so more hydrogen can be stored.^75^

Mechanical activation is also another way to increase the area of surface of the metal hydrides. Thus, their particle size reduces, and this process also produces defects in the structure of the material.^76^ Better surfaces for hydrogen absorption can be created by removing surface oxide layers through mechanical activation. Specific chemicals can also be used to remove the contaminants from the surface of the metal hydrides which is helpful in modification of the surface properties of the material.^73^ To generate structural changes and enhance hydrogen absorption/desorption kinetics, we can repeatedly expose the metal hydride to hydrogen gas at high temperatures and pressures. This mechanism also cracks surface oxides.^77^

The structural discrepancy between milled and unmilled MgH_2_ has been reported by Huot *et al.*^78^ The surface area of MgH_2_ has been reduced by milling. Accelerated H_2_ desorption kinetics, less energy of activation, and improved kinetics have been observed for milled MgH_2_ compared to unmilled MgH_2_. The formation of NaAlH_4_ by the ball milling method with titanium chloride (TiCl_3_) as a catalyst has increased the activation process.^79^ An alloy of TiFe has been proven to be better for storage of hydrogen after thermal activation.^80^ Mg-based alloys have also been activated through various activation processes.^81^

In some practical applications, there is a great need to release the stored hydrogen at lower temperatures.^82^ We can make desorption temperature low for metal hydrides through the process of activation.^73^ Catalytic doping is beneficial in lowering the activation energy of metal hydrides, as a result of which the desorption temperature of the material lowers, and they become suitable for many practical applications like portable power generation and on-board hydrogen vehicles.^51^ There are many reported metal hydrides whose desorption temperature has been lower through the process of activation. The desorption temperature of the LiBH_4_ has been lowered by ball milling and doping of transition metal in MgH_2_ has also lowered the temperature of release of hydrogen.^83^ We can control the dehydrogenation process through the activation of the metal hydrides.^84,85^ Hence, activation is essential in preparing metal hydrides for efficient reversible hydrogen storage.

### 4.3. Pressure-composition-temperature (PCT) relationships and van't Hoff plots

Pressure-composition-temperature (PCT) isotherms establish the equilibrium relationships among hydrogen pressure, hydrogen composition, and the system's temperature.^69^ In essence, it states to us, at what pressure and temperature, how much hydrogen can be absorbed and desorbed by the metal hydride. One of the most important compositional parameters is the concentration of H_2_ in the material, which affects the material's structural and functional properties, which can be determined as the weight percentage (wt%) of H_2_ in the total material. When two phases (metal and metal hydride) coexist, the curve has relatively flat regions. The length of the plateau is a measure of reversible hydrogen capacity.^86^ Hydrogen absorption and desorption increase with temperature when the equilibrium pressure rises. When hydrogen dissolves in metal at a low hydrogen concentration, a solid solution is formed. PCT curves can be used to identify the practical operating conditions of specific solid-state materials. The PCT graphical diagram fully explains the hydrogenation and dehydrogenation process.

The van't Hoff plot is a graphical illustration of the relationship between temperature and pressure at equilibrium for metal hydride. Plotting the naturally occurring logarithm of pressure against the absolute temperature provides this graph.^87^ The intercept and slope of the van't Hoff plot are used to gather information about the entropy (Δ*S*) and enthalpy (Δ*H*). This graph also allows us to compare the thermal stability of various metal hydrides. By examining the van't Hoff plot, one is also able to determine the bond strength that exists between the hydrogen and the materials.^46^

In metal hydrides, more interstitial sites are occupied by hydrogen in the crystal lattice when we increase the quantity of hydrogen.^88^ Two types of transitions are involved in the storage of hydrogen in metal hydrides. In the first phase, less hydrogen is present, and it dissolves in the metal's crystal lattice in solid-solution form. In this case, there is relatively little hydrogen present, and the metal hydride's crystal structure remains unchanged. In the second phase, a different crystal structure is formed because when a large amount of hydrogen reacts with the metal a new compound is formed. Hydrogen makes chemical bonds with metal hydrides in this phase. In this case, there is a large concentration of hydrogen, and a different crystal structure forms than pure material.^8,25,89^ The curves that display the hydrogenation and dehydrogenation must be overlapped with each other for the ideal case. For a fixed hydrogen composition and temperature, the equilibrium desorption pressure is lower than the corresponding absorption pressure. This difference, known as hysteresis, arises from internal stresses developed within the metal hydride matrix during particle growth.^90^

The schematic representation of the PCTs for absorption and desorption is presented in Fig. 1; the maximum gravimetric and the reverse capacity can be calculated from the PCTs curve.^91^ Maximum gravimetric storage capacity determines the maximum capacity to store hydrogen in metal hydride. Reversible storage capacity, meanwhile, is the amount of hydrogen released during desorption, as a function of the concentration. The reversible storage capacity is the amplitude of the plateau regions. Even if the pressure in the biphasic zone changes, the pressure at which the reaction starts is called the plateau pressure of the hydrogenation reaction (*P*_a_) and the pressure at which the reaction ends is referred to as the plateau pressure of the dehydrogenation reaction (*P*_d_).^92^ There are a number of methods to determine the plateau pressure for the reversible gravimetric capacity. The plateau pressure at the midpoint, *i.e.*, the pressure at a fixed storage capacity value (1 wt%) or at 50% of the maximum hydrogen storage capacity, is most frequently used in many methodologies related to the absorption and desorption curves.^69,93^ The plateau slope is the curve's slope that is determined at the plateau pressure:

5 Slope = Δ*P*/Δ(*H*/*M*)

where hydrogen concentration is represented by *H*/*M*, and at *P*_a_ and *P*_d_ the slope is calculated. These pressures are for the absorption and desorption of hydrogen, respectively.^94^ The logarithm of *P*_a_ to *P*_d_ ratio determined the hysteresis:6
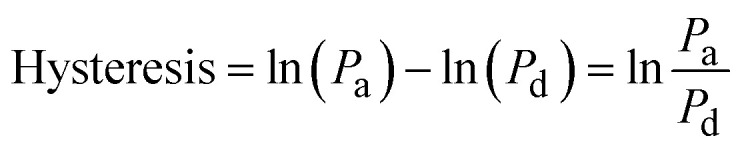


This equation describes the hysteresis for the PCT curves. It involves a natural logarithm of the ratio of absorption plateau and desorption plateau pressures, which are represented by *P*_a_ and *P*_b_, respectively, at the same H_2_ concentration. It quantifies the difference in pressure between H_2_ uptake and release by a solid-state material.

Low plateau slope values indicate that only a small amount of hydrogen can be absorbed or released within a narrow pressure range, whereas low hysteresis values suggest that hydrogen/deuterium (H/D) cycling can take place over a narrow pressure window.^95^ Based on the van't Hoff equation, plateau pressure rises with increasing temperature, a relationship that is thermodynamically expressed as:7
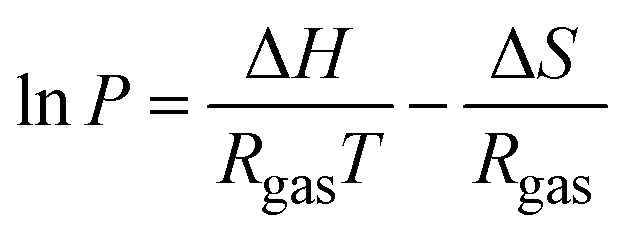


The symbols Δ*S* and Δ*H* represent enthalpy and entropy of the absorption and desorption processes, respectively, while *R*_gas_ is the universal gas constant and *T* is the absolute temperature. The values of Δ*S* and Δ*H* are negative for all the metal hydrides, indicating that hydrogenation of all these metal hydrides is exothermic. Understanding of H_2_ absorption/desorption in materials is an essential process which is explained by the use of van't Hoff equation. This equation is a relationship between the Δ*H* change and the Δ*S* change of a hydrogenation reaction with the natural log of the equilibrium pressure of a material.^96–98^ It shows a relation between the reciprocal of absolute temperature (1/*T*) and ln(*P*).

### 4.4. Resistance of gaseous impurities

For the storage of hydrogen in metal hydrides, the resistance of gaseous impurities is a critical factor because it affects the system's performance.^99^ Many impurities like water vapours, oxygen, and carbon monoxide react with metal hydride's surface, affecting the process of hydrogen absorption and desorption. This process is often called poisoning.^73^ Hydrogen uptake and release kinetics become slow because impurities occupy the active sites of material, reducing the storage capacity of hydrogen. Sometimes, degradation of the metal hydride occurs in the presence of impurities, which produce a change in the material's structure and reduce cyclic stability and storage capacity of hydrogen.^8^ In this situation, efficiency of the storage system is reduced, and more energy is needed for restoration the hydrogen from the material. We can recover the retardation and poisoning damages through cyclical exposure to H_2_.^100,101^ Hence, it is very necessary to select a appropriate metal hydride for hydrogen storage, which is less sensitive to impurities, and the surface of materials can also be modified to improve the resistance to impurities.^69^

### 4.5. Cyclic stability

For evaluating the hydrogen storage capacity, cyclic stability is a very crucial metric. It tells us that with the absorption and desorption repeated cycles of hydrogen how much the material maintains its kinetics and storage performance.^91^ This shows that over time how, much material's hydrogen capacity is loss. The rate of absorption and desorption must be consistent; this stability tells us about the changes that come in the material over time. When we store and restore hydrogen again and again, then with the passage of time, the metal hydride's volume changes. This volume change produces pulverization and cracking in the material.^69^ These physical changes can be identified with cyclic stability. For a better hydrogen system, it is expected that the material should perform many cycles of absorption and desorption. So, there is a great need to enhance the cycles of the metal hydrides. Many features like kinetics and capacity of the material are affected by this property, which is also related to the resistance of gaseous impurities. Disproportionation in the case of metal hydrides also affects the cycles of metal hydrides. When thermal diffusivity is reduced, pulverization occurs, which reduces system rates. So, cyclic stability is also affected by this.^102^

## 5. Safety

Assessing the safety of metal hydrides for hydrogen storage requires understanding their potential hazards and inherent safety benefits. As with all fuels, hydrogen utilized in automobiles has some risks that must be addressed. However, according to many popular viewpoints, hydrogen is, in many aspects, safer than other fuels typically used in contemporary internal combustion engines.^103,104^ The physical properties of hydrogen are notably peculiar: some attributes enhance the safety of hydrogen cars compared to conventional types, while others raise additional safety concerns. Hydrogen-fueled vehicles, whether powered by fuel cells or internal combustion engines, are typically considered safer than electric vehicles due to the ongoing risk of thermal runaway in batteries when subjected to overcurrent or damage.^105,106^

Hydrogen possesses an exceptional safety record. Specifically, failures of valves, filters, and pipes in the hydrogen storage system are the primary cause of incidents, although a catastrophic explosion of a hydrogen storage tank is, conversely, quite rare.^107,108^ The hydrogen vehicle's storage system is an important component that requires a comprehensive understanding and analysis of the risks associated with different hydrogen storage approaches. Safety is a crucial concern for all hydrogen storage methods.^109^

However, as will be demonstrated later, there is a wide range of metal hydrides capable of functioning under nearly ambient conditions for both pressure and temperature.^110,111^ This characteristic significantly enhances the safety of these materials relative to conventional hydrogen storage technologies. These materials beat GH2 systems as they can hold hydrogen at moderate pressures. Moreover, boil-off events in metal hydride tanks are infrequent, which minimizes hydrogen loss and makes them superior to LH2 systems.^69,112^ Conversely, many metal hydrides have pyrophoric nature, requiring consideration in tank construction to minimize explosions and flames in the event of a rupture. However, before reaching any conclusive opinions, an extensive risk evaluation must be conducted. Metal hydrides provide significant safety advantages for automobiles compared to conventional storage systems.^69^

## 6. Types of metal hydrides used for hydrogen storage

There are many different types of metal hydrides that can be used for hydrogen storage applications, and we can categorise them based on the nature of the bond and their structural properties. To improve hydrogen storage capacity, kinetics, and operational requirements, each metal hydride presents unique challenges and advantages.^51,113^ Understanding the different types of metal hydrides is important if we want to utilize them in specific applications. Each group of metal hydrides has its strengths and weaknesses in storing hydrogen. In this section, we will discuss different properties of materials and how these properties influence the potential of different metal hydrides to store hydrogen.

### 6.1. Group I and II metal hydrides

These materials are formed when the highly electropositive alkali and alkaline earth metals of groups I and II react with the hydrogen. The compound formed has an ionic character, and in these compounds, hydrogen is present as a hydride ion (H^−^).^114^ These metal hydrides can store large amounts of hydrogen per unit volume, meaning a high density of hydrogen is present in these materials. Their reactivity and stability are better due to the ionic nature of the bond.^115^ These types of materials release the absorbed hydrogen at high temperatures. Some examples of alkali metal hydrides are LiH, NaH, KH, and RbH. Whereas CaH_2_, SrH_2_, and BaH_2_ are the alkaline earth metal hydrides. Beryllium and magnesium belong to group II, but their hydrides (BeH_2_ and MgH_2_) possess covalent character. These groups of metal hydrides offer better hydrogen capacity, but their handling and safety are essential due to their high reactivity.^116^

Many metal-hydrogen compounds can be formed with light atoms like lithium, sodium, magnesium, and aluminium.^117^ Each metal atom can attract many hydrogen atoms, and they are lightweight. The storage capacities of group I metal hydrides have been extensively reported. The strong ionic bonding in these materials is responsible for their thermal stability, and we can operate them at elevated temperatures. These metal hydrides can react very quickly with the air and moisture. So, there are some challenges in their safe handling and on-board applications.^54^ LiH and NaH have been reported for the storage of hydrogen. Before storing the hydrogen, ball milling has been performed to reduce the size of the particles. LiH and NaH exhibit hydrogen storage capacities of 3.55 wt% and 1.58 wt%, respectively, at 9 bar pressure. The analysis of power density over time has proved that LiH possessed high power density after the ball milling process. The power density of LiH and NaH has been reported to be 0.025 W cm^−2^ for the long-term and 0.050 W cm^−2^ for short-term performance.^118^

A substantial amount of research exists on magnesium (Mg) and associated alloys for hydrogen storage because they possess high storage capacity by weight and are cheaper.^42^ Moreover, Mg-based hydrides (MgH_2_) have exceptional functional characteristics, including thermal resistance, vibration absorption, recyclability, and reversibility. Eqn (8) shows the reaction which occurs when hydrogen interacts with the magnesium:

8 Mg + H_2_ → MgH_2_

This chemical reaction shows that one mole of solid magnesium (Mg) is reacting with one mole of gaseous H_2_ to form one mole of magnesium hydride (MgH_2_). The space group of magnesium is *P*6_3_/*mmc*; its lattice parameters are *a* = 0.23 nm and *c* = 0.52 nm.^119^

A significant research focus has been observed on examining particular material features of Mg alloys to advance novel materials. MgH_2_ has the most significant energy. These hydrides exhibit an excellent storage capacity of 7.7 wt%, with inexpensive cost due to the plentiful availability of magnesium, with impressive reversibility.^120–123^ The primary drawbacks of MgH_2_ as a solid-state storage medium are the required elevated temperature for the discharge of hydrogen, sluggish desorption kinetics, and significant oxidative reactivity.^124,125^ The thermodynamic characteristics of MgH_2_ have been examined. The findings indicated that these materials possess elevated operating temperatures that exceed acceptable limits for practical on-board usage. The elevated thermodynamic stability of MgH_2_ leads to a significant enthalpy of desorption, which results in an unfavorable desorption temperature of 300 °C at 1 bar.^120,121,126^

Numerous attempts have been made on hydrides consists of Mg in recent years to lower the desorption temperature and expedite the dehydrogenation processes.^128^ The microstructure of the hydride may be modified to a certain degree by ball-milling with components that diminish hydride stability and by using suitable catalysts to enhance absorption and desorption kinetics.^83^ MgH_2_ before milling and after milling has been reported which are shown in Fig. 3. Before and after the milling process plateau pressure of absorption remains same. Due to formation of MgO quantity of hydrogen decreases. The milled MgH_2_ posses high desorption pressure than the unmilled MgH_2_.^78^ Magnesium hydride exhibits a substantial bonding energy of 75 kJ mol^−1^. Consequently, the dissociation of the Mg–H bond necessitates significant thermal input to induce reactivity. Empirical evidence indicates that the decomposition of MgH_2​_ commences upon reaching a temperature of 300 °C. Elevated operational temperatures present a significant challenge to proton exchange membrane fuel cell integration.^17,129^

**Fig. 2:**
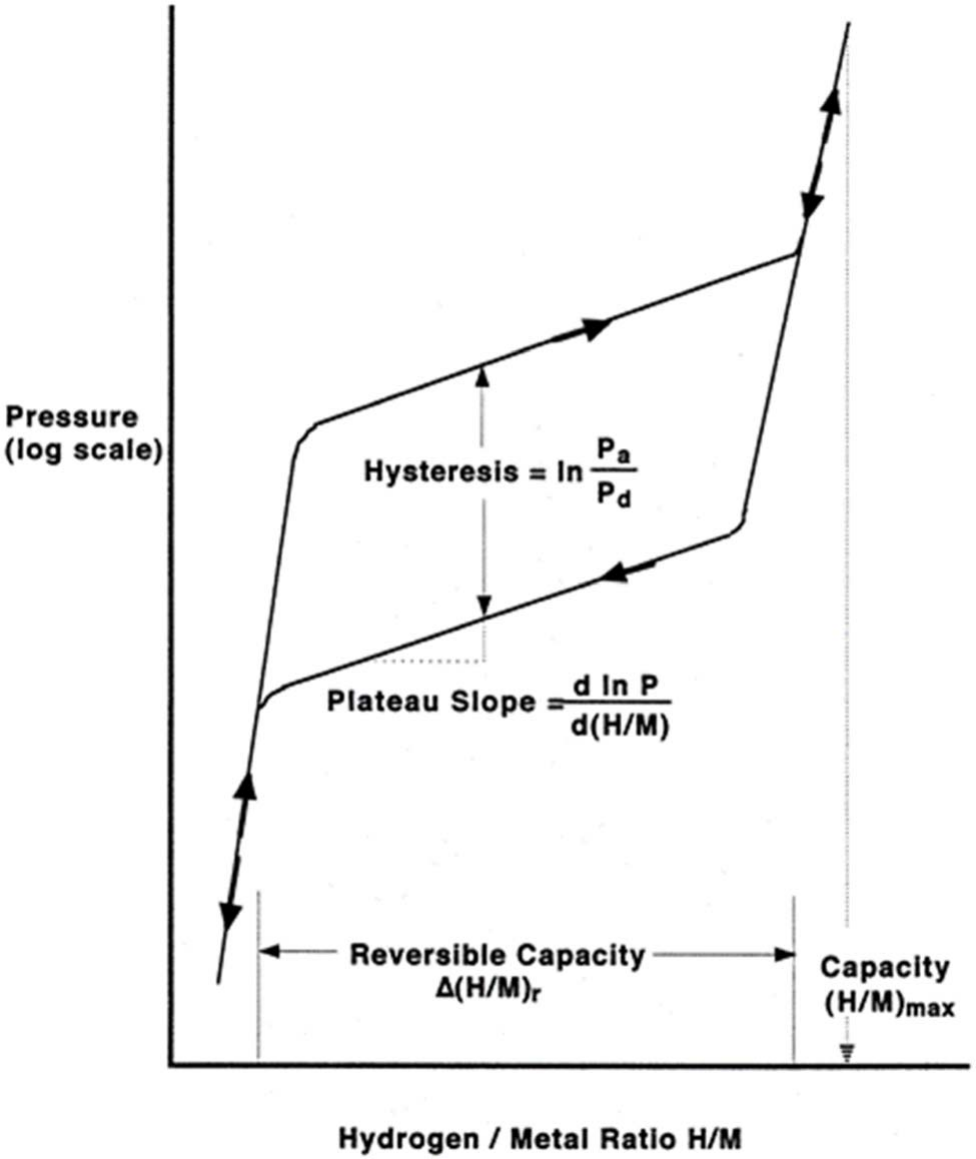
Schematic depiction of absorption and desorption PCTs. The arrow from left to right illustrates adsorption, and the arrow from right to left represents desorption. Reprinted with permission from Elsevier ref. 35.

Furthermore, magnesium hydride exhibits slow kinetic reaction rates. Research is focused on manipulating the thermodynamic properties of magnesium hydride through techniques like nanoscale engineering, alloy formation, and the creation of metastable phases. Fig. 2 shows the relationship between adsorption enthalpy and operating temperature in hydrogen storage systems.^127^

**Fig. 3:**
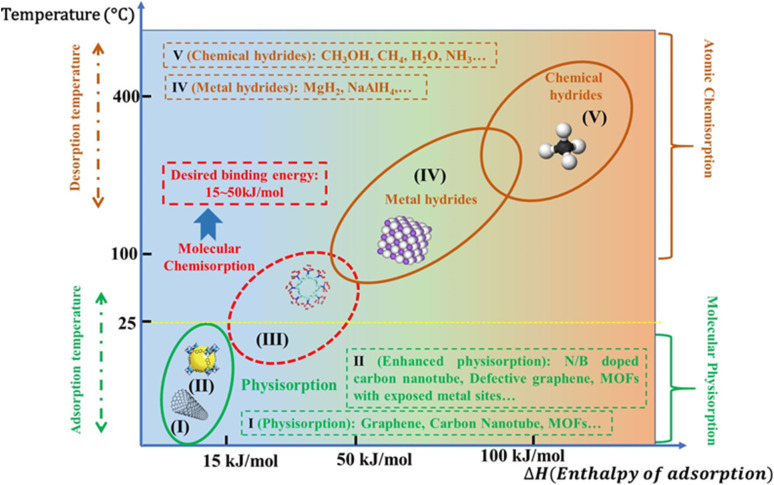
The relationship between adsorption enthalpy and operating temperature in hydrogen storage systems.^127^ Reprinted with Open access permission.

Storage of the hydrogen in group I and II metal hydrides is an effective approach.^51^ The volumetric hydrogen storage capacity of these hydrides is greater than that of compressed hydrogen storage systems because of the light packing of hydrogen in their crystal lattice.^93^

Hydrides of group I can be used in those applications where high gravimetric capacity is required with the light weight. The materials of these two groups have slow absorption and desorption kinetics that limit their usage in dynamic systems. Furthermore, they have poor reversibility and activation procedures are required to enhance hydrogen cycling. The performance of these hydrides can also be affected by the stable oxides or side products formed during the cyclic process.^51^

### 6.2. Complex metal hydrides

Complex metal hydrides are made up of light metals like lithium and sodium, whereas other metal hydrides contain heavy elements. These materials have moderate dehydrogenation pressures and temperatures, and it has been reported that complex metal hydrides possess high storage capacity.^130,131^ Borohydrides and alanates, which are complex metal hydrides belonging to group III of the periodic table, can bond with many hydrogen atoms. This bond is partially covalent or ionic. The handling of complex metal hydrides is complicated. These metal hydrides can decompose into many different elements, so it is not easy to refuel them with hydrogen. They show slow kinetics and have high thermal stability, but the hydrogen storage capacity of these materials is much less than the limit set by the United States Department of Energy.^132,133^ These issues can be reduced to some degree by adding new elements into solid-state system by substitution of anion and cation or by forming composites of hydrides. Moreover, the addition of the catalysts and nanoconfinement may also enhance the performance of the storage systems.^51,134^

Alanates or aluminohydrides are the hydrogen storage systems in which hydrogen is released when it is exposed to water. Aluminium has been widely studied in the context of metal-based complex hydrides, which are composed of alkali and alkaline earth metals. At ambient temperatures and pressures, MAIH_4_ (M = Li, Na, and K) has proved to be a better for the storage of hydrogen. These types of metal hydrides have been widely reported, and the hydrogen storage capacity of these materials has been measured to be up to 10.4 wt%.^135^ In metal hydrides, MAlH_4_ undergoes desorption by chemical breakdown. Melting of hydride is the start of this process. Three-step reaction takes place for the decomposition of MAIH_4_ and the eqn (9)–(11) show this reaction.

9 MAIH_4_(s) → MAIH_4_(l)

10
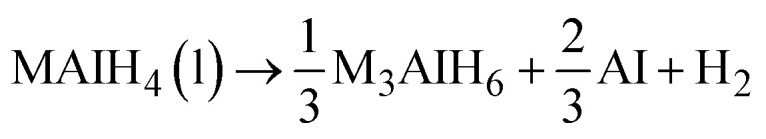
11
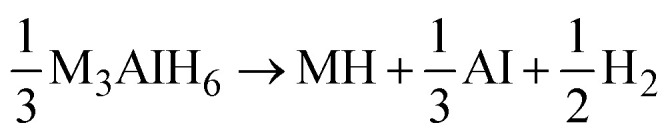


It has been noted that hydrogen storage capacities of complex aluminum hydrides containing heavy alkaline earth metals may be insufficient for automobile applications.^136^

Complex hydrides containing aluminium and sodium are sodium alanates. Sodium tetrahydroaluminate (NaAlH_4_) has been known for decades. NaAlH_4_ can be used as hydrogen storage material due to its better hydrogen storage capacity, available in bulk form and have low cost.^137^ Bogdanovic *et al.* has been noticed that we can enhanced the desorption kinetics of aluminum hydrides with the doping of titanium compounds in solid-state system and with this we can maintain the reversibility of the system at ambient conditions.^138^ It has been calculated theoretically that Na_3_AlH_6_ and NaAlH_4_ possess storage capacity of 5.9 wt% and 7.4 wt%, respectively, and the multiple steps are involved in the release of hydrogen. Firstly, intermediate compound, metallic Al, and Na_3_AlH_6_ are formed when molecular hydrogen evolves during decomposition of NaAlH_4_. Then, again metallic Al and NaH is formed with the evolution of hydrogen during decomposition of intermediate compound. The dehydrogenation of the NaAlH_4_ is defined through the eqn (12) and (13):12
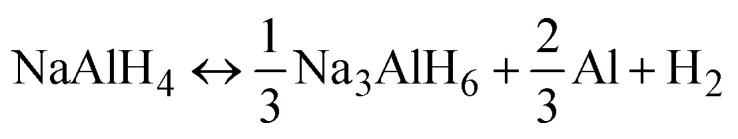
13
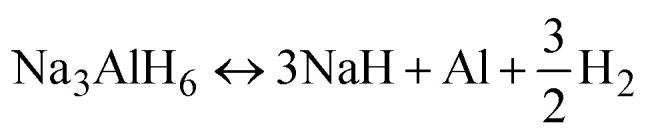


For practical applications the reversibility of reactions is a very important factor. In first step and second step 3.7 wt% and 1.9 wt% H_2_ is released, which means that 5.6 wt% of reversible hydrogen capacity is obtained during both reactions. This whole reversibility takes place at specific conditions and has slow kinetics. For the first and second reaction, the temperature is between 185 °C to 260 °C. At last, NaH decomposes at 450 °C, which is very high for the practical usage of that system.^139–141^

It has been reported by the Jensen *et al.* that the hydriding properties of sodium aluminium hydrides can be enhanced catalytically, but by doing this, the surface of the material is damaged, which decreases the cyclic capacity.^142^ The doping of TiCl_3_ has been performed for the sodium aluminium hydrides by the same group.^143^ The rate of hydriding and dehydriding processes have been increased by doping. The evolution of the hydrogen from Ti-doped NaAlH_4_ has been increased 50 times than the un-doped NaAlH_4_. This shows that doping of titanium can increase the hydrogenation and dehydrogenation processes and also enhance the system's kinetics. After the 17 cycles 5 wt% of H_2_ has been achieved with 2 mol% doping of TiN. However, with the number of cycles, decrease in the hydrogenation process has been observed.^144^ The doping of zirconium can also be used for this process. The kinetics of NaAlH_4_ has been observed to be enhanced but as a catalyst, the effects of zirconium are not as good than titanium. The detailed doping effects of titanium and zirconium in sodium alanates have been observed by Sun *et al.* by XRD technique. In the lattice parameter of materials significant changes occur after doping.^145,146^ The dehydriding kinetics can be enhanced more with the dopant rather than the cyclic effect. Gross *et al.*^147^ also observed crystal structure modification and phase transitions of doped-NaAlH_4_. It has also reported that kinetics of NaAlH_4_ can be enhanced with doping in the material. The presence of Ti is not enough for dehydrogenation reaction and specific local arrangement is necessary for this. NaAlH_4_ doped with Ti-cluster has been investigated. The condition of precursor is more important than the amount of Ti which is present in the crystal lattice. The reaction rate depends upon the size of Ti clusters, and the reaction rate can be increased with small size Ti-clusters.^148^ While Ti-doping is the common approach to the kinetic activation of NaAlH_4_, it has certain disadvantages that are not mentioned in a descriptive manner. Although the capacity of the practically realized cells is about 4.0 wt% (which is still lower than the theoretical capacity of 5.6 wt%), the use of reactive Ti-halide or Ti-alkoxide precursors requires a certain amount of the originally available hydride to be consumed during decomposition, leading to the formation of inactive Na-halide or Na-oxide by-products. This has encouraged other ways of doping, which involve lower losses, such as milling NaAlH_4_ directly with TiH_2_ or Ti powder. However, it turns out that the kinetic improvements associated with these doping routes only become apparent after approximately a dozen cycles of activation, suggesting that the doping and catalysis are distinct reactions that require Ti to diffuse into the NaAlH_4_ lattice prior to substitution at Al sites.^149,150^ This is corroborated by spectroscopic data, which show that Ti^3+^ is reduced to Ti^0^ by ball milling, and subsequently reorganizes to order in the form of Ti–Al clusters, not the as-doped precursor, before dissociating H_2_.^151^ Further cycling studies revealed that the reversible capacity stabilizes only after an initial decrease and remains largely unaffected in subsequent cycles, suggesting a balance between an increase in the conversion inefficiency of Al particles to NaAlH_4_ and further reorganization of Ti into more active Ti–Al clusters.^152^ The particle coarsening can be suppressed by confining NaAlH_4_ in Ti-functionalized nanoporous scaffolds, like MOF-74(Mg), and capacity fade is maintained at a near-constant value during repeated cycling, in line with the interpretation that the capacity fade is driven by microstructural degradation, but not by loss of catalyst activity, for bulk-doped material.^153^ Consolidated, these results indicate that although the gravimetric-capacity penalty of Ti doping can be overcome through increased kinetics, it has yet to be demonstrated to have a strong influence on the cyclic stability of the material; instead, the cyclic stability appears largely as a consequence of the evolution of Al/Na_3_AlH_6_ particles as the catalyst evolves, and thus further catalyst screening is not as direct a route to improving cyclability.^149^

Lithium alanates can also store the hydrogen in large quantities, and their H_2_ storage weight percentage is very high. Li_3_AlH_6_ and LiAlH_4_ have H_2_ weight percentages of 11.2 wt%, respectively. Unfortunately, at room temperature, hydrogen's equilibrium pressure is very high, and these materials become unstable. These metal hydrides can't be re-hydrogenated, and their decomposition is rapid.^154^ Two steps are involved in the desorption of LiAlH_4_ as given in eqn (14) and (15).

14 3LiAlH_4_ → LiAlH_6_ + 2Al + 3H_2_

15
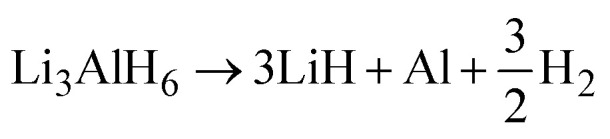


At 160 to 200 °C, the decomposition of Li_3_AlH_6_ provides 2.65 wt% of hydrogen, whereas at the same temperature decomposition of LiAlH_4_ provides 5.3 wt% of hydrogen. When the reactions are completed, 2.6 wt% of hydrogen not released and it remains present in the form of LiH and a very high temperature is needed to desorb this hydrogen.^154^ Therefore, we can't use lithium based metal hydrides for hydrogen storage because their absorption and desorption temperatures are very high, and they also have slow kinetics.

**Fig. 4:**
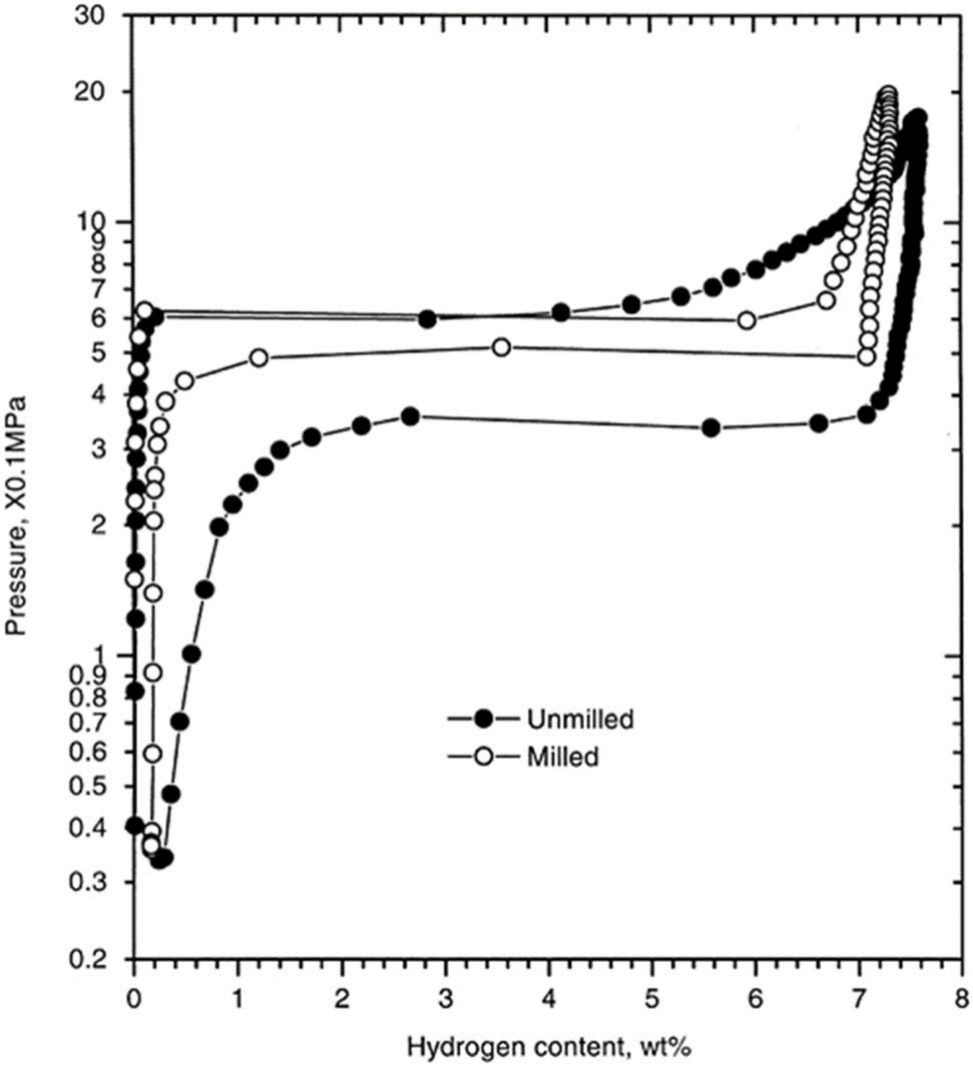
At high temperature (623 K), PCT curves of MgH_2_ before milling and after milling.^78^ Reprinted with permission from Elsevier.

The potential of KAlH_4_ to store hydrogen has also been reported. Under 10 bar of pressure and between temperatures of 250 °C to 330 °C, the hydrogen storage capacity was more than 3.5 wt% after its decomposition. It has been reported that without any catalyst, the reversible reaction is smoothly done in this case, which is very different from reactions of LiAlH_4_ and NaAlH_4_.^155^Fig. 4 shows the flowchart for classifying metal hydrides, which are primarily used for hydrogen storage applications. The poor reversibility of lithium alanate is not just an empirical fact. However, it follows from the thermodynamics of the Li–Al–H system that the thermodynamic hydrogen pressure to reform LiAlH4 directly from LiH and Al is far above what is practically attainable at moderate temperature. Decomposition will proceed effectively in one direction under normal operating conditions.^156^ An inability to find a suitable catalyst is not the reason a practically reversible bulk LiAlH_4_ system has not been achieved, nor is it the reason for the intrinsic thermodynamic mismatch in Ti-doped NaAlH_4_. If the material is nanoconfined (*e.g.*, in nitrogen-doped porous carbon, with coordination to the nitrogen sites lowering the Al–H bond dissociation energy and changing the decomposition pathway), more than 80% of the hydride can be regenerated under high pressure of hydrogen, a feat that the original literature explicitly describes as previously thought to be impossible. This is but one example of the generality of the problem: the kinetic promoter can only be effective if the thermodynamics of the process allow it to be reversible; when it is not, it is insufficient to catalyze the material; it must be thermodynamically re-engineered (*e.g.*, *via* nanoconfinement, compositing).

### 6.3. Intermetallic compounds

The compounds which are formed when interstitial sites within the crystal lattice of intermetallic compound are occupied hydrogen are known as intermetallic hydrides.^57^ They are made up of hydrogen and metallic alloy. In the crystal structure of alloy hydrogen atoms are present in interstices. These materials are good candidate for hydrogen storage, they have ability to release large volumes of hydrogen. Hydrogen can repeatedly store and released which shows that absorption and desorption processes are reversible. They are used for fuel cells, batteries and heat pumps. We can categorized the intermetallic compounds on the basis of stoichiometric ratios of alloy of constituent metals.^77^ AB-type intermetallic compounds represent a class of materials of significant interest for hydrogen storage, primarily due to their low molar mass and high gravimetric storage potential. Titanium-iron (TiFe) alloys with cesium chloride (CsCl)-like crystalline structures are an example of this category. These alloys have a hydrogen storage capacity of 1.9 wt% and reversible hydrogen uptake; at appropriate operational parameters, these materials release hydrogen.^157^ The substantial hydrogen storage capacity and rapid hydrogen adsorption/desorption kinetics determine the optimal performance of the system.^158,159^ The formation of titanium-iron hydrides occurs from the titanium-iron (TiFe) system, specifically TiFeH and TiFeH_2_, and offers advantages over lanthanum-nickel (LaNi_5_) alloy due to its lower cost.^160^ Although TiFe has potential, it has some limitations: it possesses a very low hydrogen storage capacity of 2 wt%, sluggish hydrogen sorption/desorption kinetics, and an elevated pressure of dissociation at equilibrium, which is required for complex activation.^158^ Consequently, there is a strong need to develop intermetallic hydride materials for vehicular hydrogen storage applications that exhibit better performance characteristics.

The properties of the intermetallic hydride mostly depend upon the alloy's synthesis pressure and stoichiometry. Many studies indicate that cyclic stability can be enhanced by partially replacing iron with nickel in titanium-iron alloys, which is present in nanocrystalline form.^158^ Titanium-nickel (TiNi) alloy is favourable for hydrogen uptake and has facile formability, so this material can be used for hydrogen storage. In vehicles where spatial limitations are a very important consideration, TiNi can be beneficial. This material (TiNi) exhibits a maximum hydrogen storage capacity of 1.2 wt% under controlled conditions of 400 K and 1 MPa. Due to alloys's inherent mechanical resilience, a significant challenge occurs in the synthesis of TiNi in particulate form. Additionally, the uptake of the hydrogen also reduces in this case due to the formation of the passivating oxide layers on the surface.^161,162^ The synthesis of TiNi-based composite alloys without the formation of undesired secondary phases *via* mechanical alloying is highly feasible.^163^

The intermetallic compounds that consist of a substantial proportion of LaNi_5_ and belong to the AB_5_ intermetallic category have attracted significant research attention as potential hydrogen storage media. This attention is due to the favourable operational characteristics of this material: sorption and desorption occur under mild pressure and temperature conditions, as evidenced by numerous investigations.^164–166^ The properties of both single-phase compounds and composites were investigated in several studies. Most of these materials are made by melting and mixing different metals. Key findings derived from various studies are presented subsequently.

The specific compound LaNi_5_ can absorb about 1 hydrogen atom per formula unit, giving a hydrogen storage capacity of about 1.5 wt%. However, its use is limited because the plateau pressure is low and its procurement cost is elevated. The pressure-composition isotherm shows minimal hysteresis and a plateau region. After some notable repeated cyclic process, the hydrogen storage capacity is observed to be reduced. Consequently, there are some restrictions in practical usage of these materials because they are failed to achieve the 6.5 wt% reversible hydrogen uptake which is the target of United States Department of Energy. Because these materials can be use in many commercial applications, scientists are focusing at how mixing, grinding different metals by force, or melting various metals with other metals produce change. They are also studying what results come after treating their surfaces with the carbon monoxide. Foundational investigations of LaNi_5_'s hydrogen storage capacities have been reported by Aoyagi *et al.*^167^ The impact of mechanical attrition like ball-milling has been observed, after the milling process the hydrogen storage capacity was calculated to be 0.25 wt%. Before utilization of LaNi_5_ in hydrogen storage applications, pre-treatment activation is necessary. In a prior study, Kaplan *et al.* conducted a theoretical and empirical analysis of storage of hydrogen within pristine LaNi_5_.^168^ Experimental investigations revealed that LaNi_5_ possess 1.28 wt% hydrogen capacity during hydriding process of 8.3 min. The hydriding mechanism is the interaction between LaNi_5_ and hydrogen, which is exothermic process, which demonstrated as a rapid thermal elevation. These experimental findings and computational modeling results are closely related to each other. It has been reported in many studies that controlled stoichiometric proportions of metallic additives can improve the hydrogen storage performance of LaNi_5_-based materials.^169–171^ The gravimetric storage capacities of LaNi_5_ samples with varying stoichiometric compositions have been measured to be 1.0, 1.44, and 1.32 wt%, respectively, the cyclic stability and kinetics of the hydrogen storation and restoration also improved with copper-based chemical surface modification.^169^ The surface modification of LaNi_5_ with carbon monoxide has been reported which results gravimetric storage capacity of 1.44 wt%. Conversely, Lu *et al.*^171^ adopted a twin-toll methodology on LaNi_5_, which yielded homogeneous nanocrystalline morphology and their hydrogen storage capacity is measured to be 1.32 wt%.

In addition to the previous studies, there is other published literature that should be acknowledged. Specifically, the studies by Liu *et al.*,^146^ Wang *et al.*,^147^ and Suda *et al.*^148^ reported changes in the properties of hydrogen-absorbing alloys following fluorination. Nickel-enriched layers were designed using these experimental methodologies; the objective of this research was to enhance resistance to contaminants and initial activation kinetics. The literature tells us how to design and improve containment systems if we want to use LaNi_5_ for hydrogen storage. Kikkinides *et al.*^172^ demonstrated a positive correlation between optimization of the systems and hydrogen gravimetric density for LaNi_5_-based materials. It has been observed that after refining the design parameters, storage duration increases by 60%. An analysis of LaNi_3_BH_*x*_ was conducted to investigate the hydrogen-transport mechanism. The findings showed that hydrogen atoms occupy interstitial positions; these positions are coordinated by nickel and lanthanum atoms. Furthermore, hydrogen is restricted to move along the crystal's main vertical axis due to He lanthanum-boron atomic layers. In intermetallic compounds, hydrogen storage performance is lower due to presence of boron. These compounds trap the hydrogen atoms in the material's structure and slows down the movement of the hydrogen, which keeps them away from the boron sites.^173^

Intermetallic compounds of the AB_3_ family have also been investigated as hydrogen storage media, where A represents lanthanum (La), cerium (Ce), or yttrium (Y). They have gained attention in recent years due to their excellent properties for electrochemical applications.^174^ Nevertheless, the hydrogen storage capacities of AB_3_-type intermetallic compounds are very low. For instance, amorphous hydride phase LaNi_3_H_5_ is formed when a representative of the AB_3_ structural class reacts with hydrogen during hydrogenation.^175^ However, superior hydrogen storage performance is achieved by adding manganese to LaNi_3_. The manganese substitution creates destabilization of the alloy matrix, which increases the reversible gravimetric storage capacity of the material. Using the arc-melting technique, a series of LaNi_3−*x*_Mn_*x*_ intermetallic alloys, with varying compositions denoted by *x* ranging from 0.000 to 0.400, was synthesised. The volumetric hydrogen storage capacities of these new hydrides were the same as those of the original LaNi_3_ alloy.^174^

Intermetallic compounds of the A_2_B category are formed by the combination of an alkaline earth metal (A) and a transition metal (B) and comprise a wide variety of materials. In this type, Ti_2_Ni alloys have attracted significant interest due to their excellent structural, magnetic, and hydrogen-storage properties. In Ti_2_Ni alloys, partial substitution of titanium (Ti) and Zirconium (Zr) increases the gravimetric density of hydrogen and improves cyclic stability. These substituted materials possess longer operational lifetimes and superior kinetic performance compared to the unmodified parental alloys. The structural stability of alloys decreased with substitution, leading the system toward lower temperatures.

Additionally, Ti_4_Ni_2_O_*x*_ compounds are formed by the substitution of the non-metallic constituents like O, C, and N into Ti_2_Ni alloys. The plateau pressure of hydrogen increases with compositional change and reduces the thermodynamic stability of intermetallic hydrides. Consequently, an augmentation in the intermetallic hydrides' discharge capacity is observed. Nevertheless, it is imperative to acknowledge that substantial reductions in hydrogen storage capacity are unavoidable.^176^

### 6.4. DFT insights into hydrogen storage on metals decorated materials

The interstitial hydrogen occupancy and the lattice enthalpies of the bulk classical metal hydrides control their thermodynamic behavior. At the atomic level, we can estimate whether a material have a potential to store hydrogen or not; this hydrogen storage analysis can be done with density functional theory (DFT). By calculating the adsorption energies of single H_2_ molecules to decorate metal atoms and by modeling how H_2_ molecule attaches to a decorated atom that is present on the surface of 2D slabs at the atomic scale, a direct mechanistic rationale can be provided for macroscopic thermodynamic data, which is discussed in this review.

Graphdiyne (GDY) is an allotrope of carbon that possesses sp-hybridization and homogeneous distribution of nanopores and has been reported for hydrogen storage as a host with modification in the metal adsorption sites through doping in the lattice with boron atoms.^177^ After the modification, it has been decorated with the sodium (Na) atom on both sides to enhance the H_2_ storage capacity.^178^ DFT calculations revealed that after the decoration of GDY (boron-doped) with Na, the binding energy per Na atom was 1.65 eV, and this value was greater than the cohesive energy of metallic sodium, which is 1.13 eV, indicating that Na atoms will not agglomerate (clusters will not form) on the surface of the slab and atoms will remain dispersed. In this solid-state storage system, charge-induced-dipole produces due to which five H_2_ molecules are adsorbed around each Na atom. The H_2_ capacity of this system has been calculated to be 7.73 wt%, which closely match with the H_2_ storage capacity of Mg-based hydrides (7.7 wt%). Fullerene C_60_ decorated with palladium has been studied using DFT for hydrogen storage applications. One hydrogen was reportedly adsorbed with an adsorption energy (*E*_ads_) of −0.60 eV. The value of adsorption energy for 2H_2_ adsorbed on the structure was −0.891 eV, for 3H_2_ adsorption energy was −0.877 eV, and for 4H_2_ it was −0.661 eV. All the adsorption reactions were exothermic, and hydrogen storage capacity was predicted to be 5.8 wt%, which is lower than Mg-based hydrides.^179^

Many DFT investigations have been done for light metals or transition metals decorated monolayers, such as yttrium (Y), zirconium (Zr), or magnesium (Mg). Hydrogen is adsorbed in these decorated systems when the σ-orbital of H_2_ back-donates into empty d-orbitals of the metal atoms rather than the interaction of H_2_ with metal atoms through electrostatic forces as happened in the case of Na above.

Bulk metal hydrides remain the standard for solid-state hydrogen storage, but are regulated by strong, mostly ionic/covalent M–H bonding, which results in high kinetic and thermal costs. MgH_2_ has a high theoretical gravimetric capacity of 7.6 wt%; however, the dehydrogenation of MgH_2_ begins above 300 °C under atmospheric pressure owing to a high dissociation enthalpy (76 kJ mol^−1^) and an activation energy of 160 kJ mol^−1^ after considerable efforts of catalytic and nanostructuring.^180^ Similar behavior is seen for complex hydrides such as pristine NaAlH_4_, as-milled NaAlH_4_ decomposes in three sequential steps, releasing hydrogen only above 185–230 °C in the first step and requiring temperatures above 425 °C to release the rest of the hydrogen bound as NaH, giving a usable capacity of 5.6 wt% under practical conditions.^181^ LiBH_4_ is an even more thermally demanding material with a very high theoretical capacity of 18.5 wt%, and most of its H_2_ release occurs at 400–453 °C due to the highly directed, stable covalent B–H interaction.^182^

In this context, the DFT investigations of alkali-metal decorated 2D substrates described here suggest a storage mechanism dominated by physisorption, operating under somewhat milder circumstances, but with lower absolute gravimetric capacities. Li, Na, and K-decorated buckled bismuthene (b-Bi) storing only 2.24, 2.1, and 2 wt% H_2_, with adsorption energies ranging from 0.09 to 0.19 eV, much weaker than the M − H bond strengths of bulk hydrides, and suggesting that H_2_ binding is dominated by van der Waals forces rather than the formation of hydride bonds.^183^ The two dimensional B_4_C_3_ has been reported with storage capacities (6.79, 7.19, and 5.75 wt% for 4Li@B_4_C_3_, 4Na@B_4_C_3_, and 4K@B_4_C_3_, respectively) for one-fourth metal covered B_4_C_3_ monolayers, exceeding the DOE 5.5 wt% target and approaching the practically usable capacity of NaAlH_4_, but only at desorption temperatures of 185–355 K hundreds of degrees below the onset temperatures for NaAlH_4_ or MgH_2_ dehydrogenation.^184,185^

The 2D material γ-graphdiyne decorated with Y has been reported for hydrogen storage; seven H_2_ molecules were found to be adsorbed around each Y atom with a favorable *E*_ads_ value (−0.36 eV/H_2_), and the storage capacity of this system was 6.64 wt%, and its thermal stability was also confirmed with *ab initio* molecular dynamics (AIMD) simulations. When the same γ-graphdiyne was decorated with the Zr atom, 7H_2_ molecules were observed to be bound per Zr atom. The gravimetric storage capacity for this decorated system was 7.95 wt%, and adsorption energy was reported to be greater for this system (−0.49 eV/H_2_), and AIMD simulation proved that this decorated system was thermally stable. The calculated desorption temperature of γ-graphyne-decorated Zr was 574 K with a diffusion barrier (4.05 eV) that prevents the Zr atoms from forming clusters on the surface of the slab. The hydrogen storage capacity of Zr-decorated γ-graphdiyne (7.95 wt%) is a bit more than Mg-based hydrides (7.7 wt%).^186^

The decoration of MoS_2_ with Y has been reported for hydrogen storage, and it adsorbed 8H_2_ molecules on its 2D structure, and the calculated gravimetric storage capacity of this material was 4.56 wt%.^187^ Mg with the decoration of antimony stored 20H_2_ molecules, incorporating 1 to 3 Mg atoms. The *E*_ads_ of these modeled structures were reported to be very low (0.021 to 0.064 eV/H_2_). From a minimum number of stored hydrogen to maximum loading of hydrogen, the H_2_ storage capacities have been reported to be 0.7 wt% to 8.4 wt%, which are below the standard bulk hydride values.^188^ These Kubas-type systems possess a hydrogen storage capacity of 5.9–7.95 wt%, which is close to the Mg-hydride (7.7 wt%). The *E*_ads_ values of Y and Zr-decorated graphdiyne/graphyne structures have been reported to be −0.36 to −0.49 eV/H_2_ and these *E*_ads_ values are in the range of reversible hydrogen storage.^189,190^ This *E*_ads_ range offers a thermodynamic barrier which is high enough to prevent H_2_ escape, but it is low enough to allow desorption under ambient conditions. The DFT calculations show that Kubas-type orbital binding are responsible instead of electrostatic interactions for the high storage capacity of Na-decorated systems. The molecular dynamics calculations have been reported for Y and Zr-decorated graphdiyne; desorption temperature and diffusion barrier were also calculated for Zr-decorated graphdiyne. The same evidential function is provided by hysteresis measurements and phase stability for bulk hydrides, as the desorption temperature and diffusion barrier presented for Zr-decorated graphyne, showing that a calculated *E*_ads_ is not only a fixed energy minimum but also thermally and kinetically attainable storage state.

This research provides evidence that we can also achieve the practical hydrogen storage working targets with the decoration of the surface as compared to the Mg-based hydrides. The decorated systems with different metals can improve the hydrogen storage capacity, *E*_ads_ values, and desorption temperature through Kubas-type binding and electrostatic interaction instead of occupation of hydrogen at interstitial sites within three-dimensional metal hydrides. While both metal hydride-based and two-dimensional material hydrogen storage systems possess distinct advantages and limitations, further research is required to determine the superior solid-state candidate for future technology applications.

### 6.5. Comparative overview of metal hydride classes

The hydride classes reviewed in this review are summarised in Table 1 into nine groups. Ionic hydrides such as LiH and NaH have moderate practical capacities of approximately 1.6–3.6 wt%, but the strong ionic metal–H^−^ bonding results in a high formation enthalpy, and hydrogen is only released at high temperature. In addition, their reactivity toward air and moisture calls for careful handling.^114,115,191^ Covalent MgH_2_ has a higher capacity of 7.7 wt%, but desorption onset is 300 °C because of a Mg–H bond energy of 75 kJ mol^−1^. Repeated cycling results in the formation of a passivating MgO layer that further impedes kinetics. Ball milling and catalytic doping, *e.g.*, with Ti_2_C MXene, are therefore required to improve performance.^15,16,192^ Complex alanates such as NaAlH and LiAlH_4_ are intermediate between the ionic/covalent and intermetallic extremes and offer capacities of approximately 5.6 wt% (reversible NaAlH_4_) and 11.2 wt% (LiAlH_4_), respectively, but they decompose in multiple steps, with NaH not fully decomposing until about 450 °C and only becoming practically reversible after the introduction of Ti/Zr catalytic doping.^193,194^ Complex borohydrides such as LiBH_4_ are near the practical limit of gravimetric capacity at about 18.5 wt% theoretical, but at the cost of one of the highest desorption enthalpies of any hydride class, about 74 kJ mol^−1^ H_2_, requiring temperatures above 400 °C and rehydrogenation pressures above 100 bar, so nanoconfinement and reactive hydride composites remain the principal strategies for making them usable.^195^

**Table 1:** Comparative thermodynamic and kinetic parameters of hydrogen storage of different classes of metal hydrides

Hydride class	Example	Bonding/structure	H_2_ storage capacity (wt%)	Thermodynamic properties and operating temperatures	Kinetic and reversibility limitations	Strategies	Ref
Ionic hydrides	LiH, NaH	Ionic bond	1.6–3.6	High Δ*H*; releases at high T	Slow; air/moisture sensitive	Ball milling	114,115,191
Covalent hydrides	MgH_2_	Covalent, rutile lattice	7.7	Δ*H* ≈ 75 kJ mol; 300 °C	Slow; surface oxide layer	Milling, catalyst doping	119,121,207–209
Complex alanates	NaAlH_4_, LiAlH_4_	Covalent [AlH_4_]^−^ and cation	5.6–11.2	Multi-step; up to 450 °C	Needs catalyst; capacity fade	Ti/Zr doping	135,137,210,211
Complex borohydrides	LiBH_4_	Covalent [BH_4_]^−^	18.5	Δ*H* ≈ 74 kJ mol; > 400 °C	Very slow; poor reversibility	Nanoconfinement	212 and 213
Interstitial hydrides	PdH_x_	Metallic, interstitial H	1–2	Mild, near ambient	Fast, reversible; low capacity	Alloying	196 and 197
AB-type	TiFe, TiNi	CsCl-type	1.2–2.0	High activation P	Sluggish activation	Ni substitution	157,198,199
AB_5_-type	LaNi_5_	Hexagonal CaCu_5_	1.0–1.5	Mild, low hysteresis	Low capacity; cyclic fade	Surface treatment	96 and 200
AB_2_/A_2_B	Ti_2_Ni, TiMn_2_	Laves phase	1.5–2.0	Tunable *via* doping	Stability *vs.* capacity trade-off	O/C/N doping	201–203
HEA intermetallics	TiZrCrMnFeNi	Multi-element BCC/Laves	1.6–3.6	Stable near RT	Cycling stability unproven	Entropy engineering	204–206

Interstitial and intermetallic hydrides, in contrast, operate under much gentler conditions but suffer from low gravimetric capacity due to their heavy metal composition. Bulk interstitial hydrides such as PdH_*x*_ exhibit reversible absorption and desorption near ambient pressure and temperature with fast kinetics, but the capacity is still below roughly 1–2 wt%.^196,197^ AB-type TiFe and TiNi intermetallics operate at relatively low temperatures but require high activation pressures and offer only about 1.2–2.0 wt%.^157,198,199^ AB_5_-type LaNi_5_ displays the mildest operating circumstances and lowest pressure-composition hysteresis of any class in the table. It is exactly for this reason that its capacity of just 1.0–1.5 wt% to date falls short of the U.S. Department of Energy's onboard storage targets.^96,200^ AB_2_/A_2_B Laves-phase alloys, such as Ti_2_Ni and TiMn_2_, can be tuned by considering the plateau pressure and thermodynamic stability through O/C/N doping or elemental substitution, an example of using composition engineering to move along the trade-off curve rather than breaking out of it altogether, as improvements in capacity tend to coincide with loss of structural stability.^201–203^ The only notable exception to this trend is high-entropy alloy (HEA) intermetallics, where the multi-principal-element, severely distorted BCC/Laves-phase lattices offer a denser and more diverse population of interstitial sites than in conventional intermetallics, enabling several compositions to combine near-room-temperature (RT) operation with reversible capacities of 1.6–3.6 wt%, although their long-term cycling stability is still under investigation.^204–206^

Table 1 reveals a decrease in gravimetric capacity practically monotonically from the light ionic, covalent, and complex hydride classes to the heavier intermetallic classes, whereas operating temperature and activation needs decrease in the opposite direction. No individual class of materials reviewed here meets both the DOE gravimetric target and mild operating conditions simultaneously; instead, strategies such as catalytic/dopant engineering, nanoconfinement, mechanical alloying, and compositional tuning of high-entropy systems are employed to move individual material classes toward the center of this trade-off, not to eliminate it entirely.

Table 2 shows the hydride classes with the metrics requested by the reviewer, with each data point traceable to the precise source from which it was extracted. Ionic hydrides such as LiH and NaH are decomposed only at high temperature due to their strong non-interstitial ionic bonding and reactivity to air and moisture, which limits cycling stability.^42,191^ MgH_2_ requires 73–75 kJ mol^−1^ H_2_ to desorb, which corresponds to its 300 °C onset temperature. The kinetics of MgH_2_ are still slow due to the formation of a passivating MgO layer during cycling.^121,128,207^ NaAlH_4_ is in the middle with a first desorption step of about 37 kJ mol^−1^ H_2_ and a second of about 47 kJ mol^−1^ H_2_. It only became practically reversible after Bogdanović’s Ti/Zr-doping work, and that is the main source of its cycling behaviour.^145,214,215^ LiBH_4_ is at the most thermodynamically demanding end of the table with a desorption enthalpy of 74 kJ mol^−1^ H_2_ requiring > 400 °C and > 100 bar rehydrogenation pressure, giving it the least reversibility of the classes surveyed.^182,216^ By contrast, interstitial and intermetallic hydrides exhibit far lower desorption enthalpies and less demanding operating conditions: bulk PdH_*x*_ desorbs at around 37 kJ mol^−1^ H_2_ at near ambient temperature with rapid and highly reversible kinetics,^8,217^ TiFe requires 28–35 kJ mol^−1^ H_2_ across its two-step hydride formation but suffers from difficult activation and oxide passivation,^158,218^ and LaNi_5_ desorbs at around 31 kJ mol^−1^ H_2_ with negligible hysteresis but a well-documented capacity fade on repeated cycling.^167,168,218^ The AB_2_/A_2_B Laves-phase alloys show that this enthalpy can be further tuned by doping with non-metals, but at the cost of structural stability.^168,219^ Again, high entropy alloys are a class that stand out, combining relatively low desorption enthalpies with fast kinetics and operation close to ambient conditions, although their long-term cycling behavior is being established in the literature.^209,210^ In total, the table confirms that the desorption enthalpy is the single most predictive parameter for both the operating temperature and kinetic behaviour for all classes and that no class in this comparison combines a low enthalpy with proven long-term cycling stability.

**Table 2:** Comparison of operating temperature, desorption enthalpy, kinetics, cyclic stability, advantages, and limitations of different metal hydrides

Material system	Operating temperature	Desorption enthalpy (kJ mol^−1^ H_2_)	Kinetics	Cycling stability	Advantages	Limitations	Ref.
Ionic hydrides (LiH, NaH)	Decomposes only at high temperature (>500 °C)	High (non-interstitial ionic bonding)	Slow	Poor; degrades on exposure to air/moisture	Simple synthesis; strong, stable bonding	High-temperature release; reactive, hard to handle	42 and 191
Covalent hydride (MgH_2_)	300 °C onset (1 bar)	∼73–75	Slow, oxide-layer limited	Improves after ball milling/doping	Cheap, abundant Mg	Slow kinetics; surface MgO passivation	121,128,207
Complex alanate (NaAlH_4_)	185–260 °C (catalyzed two-step); NaH decomposes ∼450 °C	∼37 (step 1)/∼47 (step 2)	Slow uncatalyzed; much faster with Ti/Zr doping	Reversible for many cycles once catalyzed	Catalytic doping makes it reversible near-practically	Multi-step decomposition; final NaH step still high-T	145,214,215
Complex borohydride (LiBH_4_)	> 400 °C	∼74	Very slow	Poor; requires >100 bar/>400 °C to rehydrogenate	Very high theoretical capacity	Extreme thermal stability; stable intermediates block full release	182 and 216
Interstitial hydride (PdH_*x*_)	Near ambient	∼37 (bulk Pd)	Fast	Excellent, minimal fade	Fast, fully reversible near room temperature	High material cost; not scalable	8 and 217
AB-type intermetallic (TiFe)	Mild, near ambient, but high activation pressure needed	∼28–35 (two-step, FeTiH/FeTiH)	Slow activation	Sensitive to surface oxide/impurities	Lower cost than LaNi_5_; mild sorption/desorption	Difficult initial activation; oxide passivation	158,162,218
AB_5_-type intermetallic (LaNi_5_)	Ambient	∼31	Fast	Capacity decreases after repeated cycling	Minimal pressure hysteresis; mild conditions	Cyclic capacity fade; La/Ni cost	167,168,218
AB_2_/A_2_B intermetallic (Ti_2_Ni, TiMn_2_)	Tunable *via* doping	Reduced by O/C/N substitution (relative to undoped)	Moderate	Improves with elemental substitution	Thermodynamics tunable by composition	Structural stability trades off against tuning gains	168 and 219
HEA intermetallic	Near ambient; several need no activation	Low-moderate, composition-dependent	Generally fast	Promising; long-cycle data still limited	Combines near-ambient operation with comparatively higher capacity	Long-term cycling and scale-up not yet fully established	220 and 221

## 7. Mechanism of hydrogen storage in metal hydrides

**Fig. 5:**
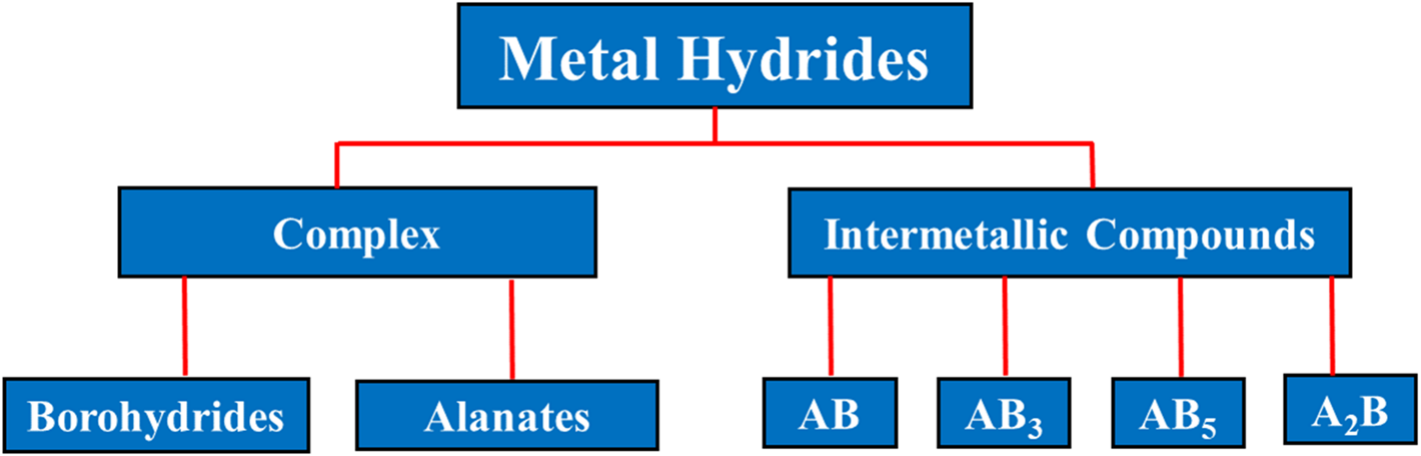
Classification of the metal hydrides.

Understanding the mechanisms responsible for hydrogen storage in metal hydrides is essential for optimizing their performance in various applications. In this section, we will discuss the processes involved in hydrogen interaction with metal lattices. Absorption is the process of storing hydrogen gas in the metal crystal lattice.^51,222^ In this process, hydrogen molecules form a chemical bond with metal atoms, which is exothermic. Whereas desorption is the reverse of the absorption in which hydrogen is released from the metal hydride, and this process is endothermic. Fig. 5 shows the absorption and desorption of hydrogen in metal hydride storage tank.^223^

It is necessary to understand hydriding and dehydriding if we want to know how metal hydrides function for hydrogen storage. When metal alloys absorb hydrogen gas, this process is called hydriding. This process is a more specific form of absorption in which hydrogen reacts with metal at low temperature or high pressure to form a stable compound. Hydrogen molecules are absorbed in metal, and then the dissociation of hydrogen molecules occurs, and hydrogen atoms are formed. These atoms form chemical bonds with the metal atoms and occupy interstitial sites. Storage of hydrogen is dependent on the hydrogen's pressure, temperature, and type of metal. When metal hydride is formed, the crystal structure of metal changes and the compound formed has higher hydrogen storage densities than liquid hydrogen.^8^

In the dehydriding process, hydrogen is removed from metal hydride. When we provide a high temperature to the system, hydrogen atoms gain energy and recombine to form hydrogen molecules, and the hydrogen is released from the metal's crystal structure.

**Fig. 6:**
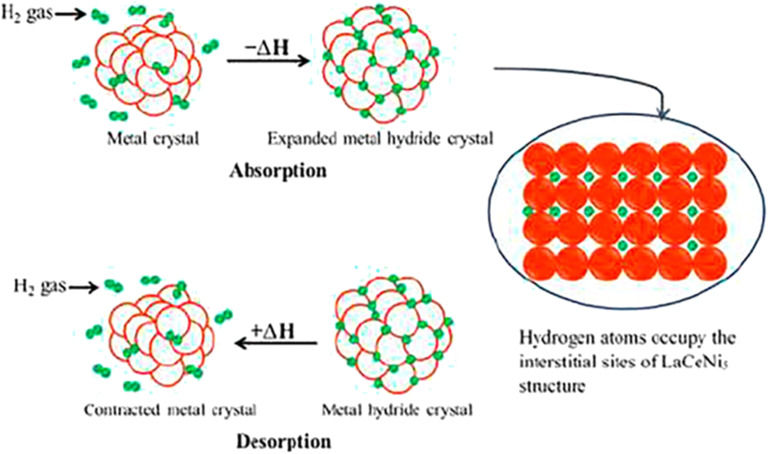
Mechanism of absorption and desorption of hydrogen in metal hydride storage tank.^223^ Reprinted with Open access permission.

**Fig. 7:**
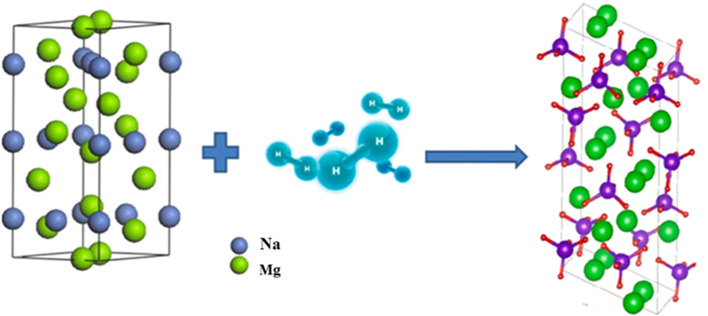
Hydriding and dehydriding process of Mg_2_Ni intermetallic system.^224^ Reprinted with permission from Elsevier.

The intrinsic properties of metal hydrides, pressure, and temperature affect the dehydriding rate.^8^ For practical applications, controlling the hydriding and dehydriding characteristics is essential, and this is a key focus of ongoing research. Fig. 6 shows the Mg_2_Ni intermetallic system, its hydriding and dehydriding.^224^ The hydrogen uptake leading to hydride phase formation is governed by a series of kinetic barriers, including subsurface penetration and bulk diffusion. Following the initial absorption of hydrogen molecules, a surface hydride layer is posited to form, significantly reducing the hydrogen diffusion rate. For example, the diffusion coefficient in magnesium hydride (MgH_2_) at 350 °C is approximately 1.5 × 10^−16^ m^2^ s^−1^, substantially lower than the 1 × 10^−8^ m^2^ s^−1^ observed in pure magnesium at the same temperature.^225^ The potential of Ti_2_C MXene in order to promote the catalytic effect and dehydrogenation of MgH_2_ has been investigated.^137^ The schematic representation of the mechanisms governing hydrogen release *via* Ti_2_C in catalyzing the dehydrogenation of MgH_2_ is shown in Fig. 7.^226^

## 8. Parameters which effect the hydrogen storage

The potential of metal hydrides to store hydrogen is influenced by two main parameters, each of which plays a very important role in the performance of materials. In the following section, we will study how particles' activation energy and size affect the hydrogen storage kinetics.

### 8.1. Size of particle

The investigations conducted by Mulas *et al.*^195^ and Arico *et al.*^228^ have demonstrated that nanoscale structures exert a substantial influence on the sorption and desorption of hydrogen. In particular, these configurations reduce the diffusional path length while simultaneously increasing the interfacial area between the electrode material and the electrolyte. This phenomenon allows and supports the migration of hydrogen atoms towards the electrode's interface. In addition, it raises the atomic density at grain boundaries and enhances the hydrogen diffusion coefficient. Different approaches, such as hydrolysis, sol–gel synthesis, combustion synthesis, high-energy mechanical milling, mechano-chemical activation, and rapid solidification by melt spinning, are employed to produce amorphous and nanocrystalline phases.^229–231^ Mechanical alloying is one of many processes that produce nanostructured materials using a relatively inexpensive, less acoustically rigorous approach.^232^

**Fig. 8:**
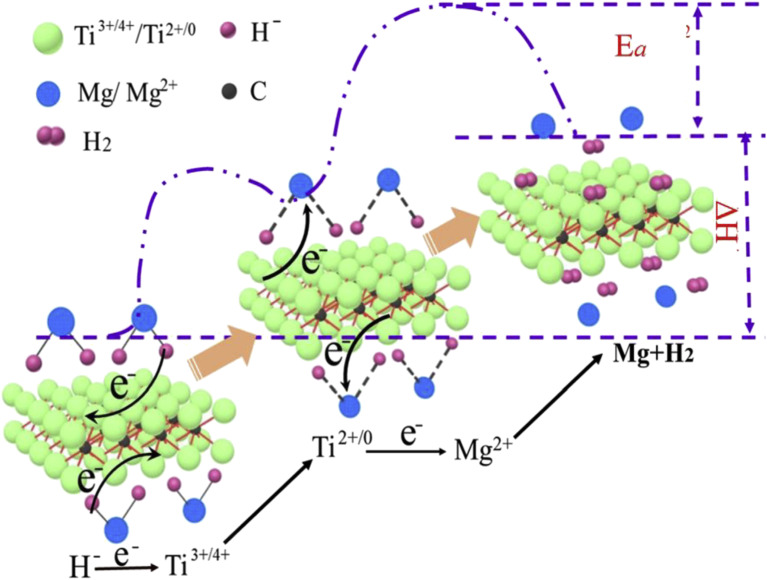
Schematic representation of the mechanism governing dehydrogenation of MgH_2_ in the presence of Ti_2_C MXene.^227^ Reprinted with permission from Elsevier.

**Fig. 9:**
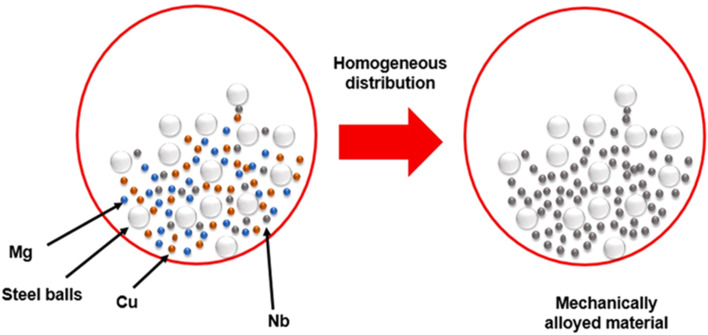
Mechanical alloying of the metal. The steel balls are represented by orange.^224^ Reprinted with permission from Elsevier.

Mechanical alloying (MA) is a technique to process solid form particulate materials (Fig. 9). The approach utilises high-energy ball milling to pulverise and recombine the powder constituents for microstructural refinement. The most common operational behaviour is the production of heat through collisional interactions between milling media, which leads to a continued chain reaction of particle breakage and agglomeration, also accompanied by alloy constituents interdiffusion at the impact interfaces. Fig. 8 shows the MA process of metal.^224^

However, the large difference in their fusion temperatures gives rise to a metallurgical challenge for conventional synthesis techniques for Mg–Ti alloys. Importantly, the production of nanostructured materials is challenging using conventional methods, especially compared with large-scale processes for producing Mg–Ti systems. Due to differences in their melting temperatures and the very limited solid solubility of titanium in magnesium, MA was considered the best method for producing nanostructured Mg–Ti alloys.^233^ Mechanical alloying is a general method to reduce the activation energy barrier for hydrogen sorption and desorption. Important factors which enhanced the storage of hydrogen are more surface area, decreased particle size, less activation time, lower activation pressure and temperatures, and faster kinetics. It has been noticed that MA satisfies all the above conditions and has attracted much academic interest.^234^

### 8.2. Activation energy

In the frame of hydrogen absorption/desorption phenomena, Myer noted that activation energy is an important parameter. The activation energy is the kinetic barrier first described by Svante August Arrhenius in 1889, the lowest energy threshold for the rupture of chemical bonds and the activation of atomic and molecular species in the forward direction of chemical reactions.^235^ The evaluation of activation energy is performed *via* Kissinger analysis, utilizing differential thermal analysis. The activation energy is conventionally denoted as *E*_a_ as per eqn (16):16
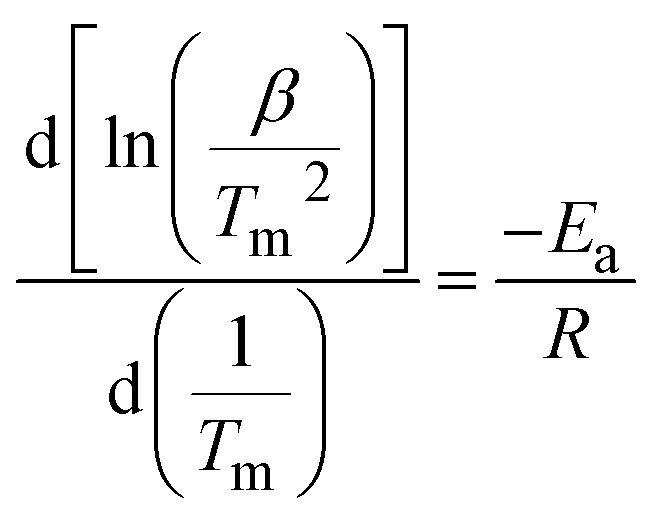
In this equation, *β* is the heating rate (in degrees celsius per minute), *T*_m_ is the temperature at which hydrogen evolution is maximal (in Kelvin scale), *E*_a_ the activation energy (in kilojoules per mole) and *R* is the universal gas constant.

The MA methodology helps in obtaining the minimum activation energy. The method involves the introduction of abundant structural defects and introducing some localized stress fields, thus creating increased atomic diffusivity. In addition, the fast atomic transport facilitates the quick homogenization, which results in fast reaction kinetics. The generation of novel surfaces with distinctive compositions further expedites the diffusion phenomena. Venkateswari *et al.*^236^ studied the structure of Mg_2_Ni in various morphologies. In particular, the samples of Mg_2_Ni were processed by MA for 20, 30 and 50 hours. The activation energy, *E*_a_ (64 kJ mol^−1^), the coefficient of diffusion of hydrogen and the hydrogen discharge capacity (474 mAhg^−1^) of Mg_2_Ni are lower than the crystalline form, which is 95.77 kJ mol^−1^, and in the amorphous form (175.86 kJ mol^−1^), respectively. The capacity retention of the nanostructured material is better (73%) than that of the crystalline and amorphous counterparts. As the milling is continued for a longer period, the number of cold welding events increases and leads to particle aggregation, and this in turn causes a reduction in the rate of hydrogen release.

The researchers have studied the combination of different alloys, including metal species like Mg_1.75_Nb_0_._25_Ni, Mg_0.8_M_0.2_Ni (M: Al, B, Ti, and Zr), Mg_2_Cu, Mg_2_Fe and Mg_6_Pd, as well as the addition of oxide phases like Nb_2_O_5_, Cr_2_O_3_ and Fe_3_O_4_. Several alloying substitutions have been noted to improve the surface properties and electrochemical reaction rates in alkaline electrolytes. The U.S. Department of Energy has adopted a criterion for onboard vehicles that requires a minimum gravimetric capacity of 6.5 wt% and a minimum volumetric capacity of 62 kg H_2_/m^3^ for the selected metal hydride material.

Alloying substitutions have been observed to enhance the surface properties and electrochemical reaction rates in alkaline electrolytes. The United States Department of Energy has established target metrics for onboard vehicle hydrogen storage, specifying a minimum volumetric capacity of 62 kg H_2_/m^3^ and a gravimetric capacity of 6.5 wt% for the chosen metal hydride material.^237,238^

## 9. Hydrogen storage applications of metal hydrides

Metal hydrides can store and release hydrogen and can be used in many applications. This section discusses various applications in which these materials can be used.

### 9.1. Stationary and transportation applications

Metal hydrides offer various advantages for both stationary and transportation applications. These materials can store hydrogen safely and have high volumetric densities. They are safer than compressed or liquid hydrogen storage systems. Metal hydrides bind the hydrogen chemically at low pressure and reduce the leakage and explosion risk. This safety feature of those materials is very attractive for public safety.^239^ Metal hydrides can be used in stationary applications in hospitals, telecommunication facilities, and data centres. Specifically, metal hydride storage tanks can be used in power systems for backup electricity, while metal hydride thermal storage units or heat pumps can manage the heat.^240^ Early prototype devices show the potential of these devices for thermal regulation and efficient energy storage in critical environments. In the future, we can use them in industries where a consistent hydrogen supply is required, such as electronics and chemical manufacturing. They can be used in backup power systems. These materials can store hydrogen, which is produced by wind and solar power.

Metal hydrides are better candidates for fuel cell electric vehicles (FCEVs).^241^ The problem is only that their weight is higher, and they have slow kinetics. The researchers are focusing on improving their kinetics. They can be used in trucks and buses where there is less space, and they are also safer. We can also use them in aviation and maritime applications. Metal hydrides are used in submarines, proving their feasibility in mobile applications.^242^

### 9.2. Challenges and future direction

There are some challenges in the usage of metal hydride-based hydrogen storage systems; addressing these challenges is crucial for realizing the potential of this technology. The weight of the materials is one of the key factors. The present metal hydrides are heavy, this condition influences the transportation range and efficiency.^243^ The development of lightweight metal hydrides should be emphasized. More research is needed in the future in the field of new metal hydrides that have higher storage capacities and lower weight. There are also important issues of slow kinetics and heat management of such storage systems.^224^ There is also a great need to develop advanced characterization techniques to understand the behaviour and properties of metal hydrides.^171^ Sustainability and recyclability of these materials need to be addressed for improved hydrogen storage performance. Moreover, the comparatively low atomic mass of beryllium compared to other light metals has prompted new computational studies of beryllium-based hydrides and beryllium-decorated nanostructures as potential high-capacity pathways for hydrogen storage. Density functional theory and neutron-scattering studies indicate gravimetric hydrogen densities of roughly 18.3 wt% for beryllium hydride, BeH_2_, and 15.9 wt% for lithium-beryllium hydride, LiBeH_3_, compared to magnesium and aluminum-based hydrides.^244^ The hydrogen in LiBeH_3_ is highly mobile at around 265 K, close to ambient temperature. First-principles calculations on lithium- and boron-decorated beryllium hydride monolayers have shown reversible gravimetric capacities of up to 14.5 wt% H_2_ with adsorption energies compatible with near-ambient cycling.^245,246^ In addition, related first-principles work on beryllium borohydride [Be(BH_4_)_2_], isolated beryllium clusters, and a beryllium-based metal–organic framework has also demonstrated thermodynamically favorable hydrogen uptake and release.^247^

Nevertheless, the beryllium-based systems have to cross a barrier that is independent of their thermodynamics for their theoretical promise. Beryllium and beryllium components are human carcinogens of group 1. Epidemiological research has revealed that exposure to beryllium metal, certain beryllium alloys, and beryllium hydroxide may develop lung tumors even at low exposure levels.^248^ Even researchers calculating Be(BH_4_)_2_ from first principles have acknowledged that beryllium-based hydrides would likely not be used for large-scale hydrogen storage owing to their toxicity. Thus, beryllium chemistry is a useful theoretical benchmark for the limits achievable by minimizing atomic weight in gravimetric capacity, but is not considered a viable engineering pathway for practical hydrogen storage; future work in this direction is likely to remain limited to fundamental computational and materials-science studies rather than applied device development.

#### 9.2.1. Current gaps and emerging materials for future outlook

Solid state hydrogen storage is at a critical stage, although a large performance gap still exists. It is challenging to identify a material class that fits the gravimetric and volumetric storage requirements under mild ambient conditions for on-board applications. Current researches are confined to optimization situations where advances in either kinetic or thermodynamic reversibility are nearly often linked with large weight penalties or structural deterioration. However, to bring solid-state systems from the laboratory bench to scalable industrial applications, research should move beyond successive iterations of modifications of legacy systems and actively seek to address the mechanistic blind spots, design new multi-component catalyst scaffolds, extend the operating range of high-entropy lattices, and capitalize on computational screening of novel, low-dimensional materials. In the case of bulk-doped complex systems, such as titanium-catalysed NaAlH_4_, the transition metal undergoes a shift in its redox state, and the active transition-metal atoms form local active clusters to break H_2_ molecules.^145,214,215^*In situ* structural characterisation of the spatial distribution of these clusters during continuous high-rate cycling and the transient boundaries that link intermediate phases, however, is inevitably not fully understood due to the lack of *operando* structural characterization. In contrast, covalent storage systems based on magnesium chemistry tend to form a surface hydride film very rapidly, which strongly hinders subsequent bulk hydrogen diffusion by several orders of magnitude relative to pure magnesium. Little is known about how atoms travel across the passivating oxide (MgO) interfaces that form at the sites of thermal spikes during high-pressure reloading of light metals.^249^ In addition, a common physical model to explain and quantify the effect of long-range lattice strain arising from non-stoichiometric occupancy on macrostructural pulverisation has not yet been identified in interstitial and intermetallic compounds. Going forward, further efforts should be directed towards more catalytic screening to investigate methods for inhibiting microstructural coarsening without compromising the density of active material. Standard kinetic activation processes require using a fraction of the starting host matrix with reactive transition-metal halides or alkoxides, thereby introducing stable, inactive by-products, referred to as dead weight, that penalise net gravimetric capacity. There is a need to investigate new pathways for doping that do not involve catalyst destruction, such as co-milling with passivated transition-metal clusters or sub-nanometre metallic wires. Confining complex hydrides in functionalized nanoporous scaffolds can physically inhibit the long-range movement and coarsening of metallic grains, which are the primary causes of cyclic capacity fade, and can keep capacity values remarkably stable over long cycling. Furthermore, as a fast electron-conduction pathway and compliant mechanical barrier to accommodate severe lattice volume changes. High-Entropy Alloys (HEAs) offer a new way to overcome the limitations of binary or ternary intermetallics by combining multiple principal elements, such as TiZrCrMnFeNi, to exploit high mixing entropies for stabilising severely distorted body-centred cubic (BCC) or Laves phases. This extreme structural distortion creates a much more diverse population of interstitial asymmetric sites, which can provide some formulations with a very ‘non-trade-off’ type curve of reversible storage capacity and operating temperature (near room temperature).^250^ The binding energy of interstitial hydrogen can be fine-tuned by controlling the local chemical environment, which is continuously varying, without using macroscopic element substitution (entropy engineering). Although the theoretical potential is clear, the cycling stability of HEA systems has not yet been demonstrated with hundreds of continuous operational cycles; the degradation mechanisms remain unclear, and resistance to impurity poisoning has not yet been demonstrated. Developing scalable, cost-effective synthesis routes that avoid the formation of segregated secondary crystalline phases is a major engineering challenge. The advances in bulk material modification have been paralleled by a completely new paradigm: the substitution of macroscopic three-dimensional interstitial absorption with coordinated surface adsorption on metal-decorated two-dimensional substrates, which has opened the door to first-principles computational modelling *via* density functional theory (DFT). DFT calculations can be used to screen the exact adsorption energy of the molecular hydrogen around the isolated metal centres that are decorated on ultra-lightweight 2D matrices. In other words, transition metals, such as yttrium or zirconium, are decorated on the gamma-graphdiyne scaffolds, instead of forming rigid ionic/covalent bonds that would lead to the destruction of the carbon network, to obtain the optimum adsorption energies, which would stop the volatiles from escaping at ambient temperature and allow them to be desorbed with a small amount of energy.

Ultra-lightweight configurations have been screened recently for first-principles research to attain gravimetric efficiency, such as sodium adorned boron-doped graphdiyne as a host.^177^ Light beryllium monolayers have not been considered for practical application due to excessive toxicity, but the theoretical limitations of 14.5–18.3 wt% and highly mobile kinetic profiles.^244^ The main challenge for computational screening in the future is to identify structurally stable 2D monolayers that exhibit these extremely high capacities and gentle desorption kinetics. Moving forward, future modelling efforts need to go beyond simply exploiting ground-state energy minima and require a much broader set of *Ab Initio* Molecular Dynamics (AIMD) simulations to compute diffusion barriers, with the requirement that the decorated metal complexes do not agglomerate during high-temperature cycling and do not form surface clusters. Scalable and wet-chemical synthesis of such surface models is crucial for the next generation of materials engineering.

### 9.3. Advancements in emerging paradigms and advanced characterization frameworks

Recent advances have brought strong paradigms into the field of solid-state hydrogen storage that overcome the historical compromises of this field by utilizing multi-component material systems, advanced computational intelligence and real-time atomistic tracking. High-entropy hydrides (HEH) are a novel, emerging family of materials based on a large composition space and multi-principal elements, resulting in significant lattice distortion. The distortions lead to a very high density of asymmetric interstitial defect sites, resulting in very tunable thermodynamics and excellent structural stability for long cycling. Coordinated with these bulk structural modifications, nanoconfinement approaches that involve embedding complex or reactive light-metal hydrides in functionalized nanoporous carbon or metal–organic framework (MOF) structures overcome bulk diffusion barriers and suppress microstructural coarsening.^251^ In these systems, the active phases are physically confined to the nanoscale, resulting in reduced phase separation, reduced kinetic activation energies and greatly enhanced cyclic stability. The deployment of machine-learning-enabled materials discovery and computation-driven catalyst design is driving the rapid discovery of these advanced configurations.

A variety of high-throughput machine learning algorithms trained on large quantum mechanical datasets can quickly evaluate millions of alloy compositions and 2D substrate interfaces to predict accurate hydrogen adsorption energies and stability of these compounds prior to experimental synthesis.^252^ This is directly complemented by first-principles, computation-guided catalyst design, involving the use of DFT and AIMD to engineer zero-loss catalyst interfaces, optimise Kubas-type back-donation interactions, and model diffusion pathways across phase boundaries. The advanced *operando* characterization techniques are essential to validate these computational models and understand the exact kinetics of these materials under operation. With real-time *in situ* X-ray diffraction, neutron vibrational spectroscopy, and environmental TEM, researchers can now follow the transient evolution of phase transformations, map the distribution of local atomic strain, and observe active catalytic cluster migrations within realistic temperature and pressure envelopes.^252^ These integrated computational, structural, and real-time analytical metrics set the current state of engineering for high-performance, scalable solid-state hydrogen storage systems.

## 10. Summary

Hydrogen is regarded as a sustainable and clean energy carrier and may be able to help in solving problems which the world is facing, like fossil fuel depletion and environmental pollution. The use of hydrogen on a larger scale, however, strongly depends on safe and efficient storage technologies with high hydrogen gravimetric capacities. In the hydrogen storage systems, solid-state storage systems, based on metal hydrides, are considered as promising storage systems due to their high volumetric storage density, operational safety, and better reverse hydrogen storage capacity. Hydrogen storage in the crystal lattices of metals or metal alloys, which can form hydrides, is due to chemical or interstitial bonding. The moderate temperature and pressure of metal hydrides make these systems safer. The release of hydrogen from the material is a purely endothermic process, thus creating an inherent safety mechanism. Hydrogen is desorbed with additional heat input that is continuously supplied, which prevents uncontrolled hydrogen leakage. The properties render metal hydrides as very promising components of fuel cell vehicles, stationary energy and portable power systems.

The review article highlights many different types of metal hydrides, such as covalent, ionic, metallic (interstitial), complex hydrides and intermetallic compounds. The kinetic and thermodynamic properties of the metal hydrides are different in each category. Covalent and ionic hydrides are more stable and have stronger bonds, whereas interstitial hydrides exhibit faster reactivities and improved reversibility. Complex hydrides, such as borohydrides and alanates, consist of light-weight elements which possess high gravimetric storage capacities but have poor reversibility and high desorption temperatures. Magnesium-based hydrides have been proven to exhibit considerable potential because of their low cost and high hydrogen storage capacity of 7.7 wt%. But their usage is quite restricted because of the high operating costs and slow kinetics. The performance of metal hydrides is affected by several parameters such as reaction kinetics, activation process, PCT behaviour, resistance to impurities and cyclic stability. The high thermodynamic stability gives rise to high temperature, and slow absorption or desorption is a major problem. Other factors such as pulverization, impurity poisoning and degradation also impact the long-term performance. Thus, lowering the activation energy, increasing cyclic stability and improvement of kinetics are important for practical applications.

Alloy design, mechanical milling, and catalyst doping have led to important advances in developing nanocomposites and high-entropy alloys. These all lead to reduced diffusion path-lengths, increased reversibility, a change in thermodynamic properties and better diffusion of hydrogen. Surface modifications through protective treatment improve resistance to gas impurities and cyclic life. Heat management, small gravimetric capacity and high system weight are still challenges compared to the mobile application's targets. Improvements of the durability, optimization of light hydride system design, optimized thermodynamics and faster kinetics are recommended for future research. Novel materials must be developed using computational modelling of materials along with innovative methods of synthesis.

Metal hydrides represent a very safe and efficient method for storing hydrogen. Despite many difficulties associated with solid-state metal hydride-based systems, innovations in the fields of nanotechnology and materials science will enable continuous improvements in their performance. By integrating and optimizing such systems, it is possible to contribute significantly to the transition to clean energy systems based on hydrogen.

## Conflicts of interest

The authors declare that they have no known competing financial interests or personal relationships that could have appeared to influence the work reported in this paper.

## Data Availability

This is review article, so data sharing is not applicable as no new primary datasets were generated or analyzed during the current study. All data, insights, and structural values discussed and synthesized within this manuscript are sourced from the literature and fully acknowledged *via* references within the text.
